# Structure, stability and specificity of the binding of ssDNA and ssRNA with proteins

**DOI:** 10.1371/journal.pcbi.1006768

**Published:** 2019-04-01

**Authors:** Arumay Pal, Yaakov Levy

**Affiliations:** Department of Structural Biology, Weizmann Institute of Science, Rehovot, Israel; Rutgers University, UNITED STATES

## Abstract

Recognition of single-stranded DNA (ssDNA) or single-stranded RNA (ssRNA) is important for many fundamental cellular functions. A variety of single-stranded DNA-binding proteins (ssDBPs) and single-stranded RNA-binding proteins (ssRBPs) have evolved that bind ssDNA and ssRNA, respectively, with varying degree of affinities and specificities to form complexes. Structural studies of these complexes provide key insights into their recognition mechanism. However, computational modeling of the specific recognition process and to predict the structure of the complex is challenging, primarily due to the heterogeneity of their binding energy landscape and the greater flexibility of ssDNA or ssRNA compared with double-stranded nucleic acids. Consequently, considerably fewer computational studies have explored interactions between proteins and single-stranded nucleic acids compared with protein interactions with double-stranded nucleic acids. Here, we report a newly developed energy-based coarse-grained model to predict the structure of ssDNA–ssDBP and ssRNA–ssRBP complexes and to assess their sequence-specific interactions and stabilities. We tuned two factors that can modulate specific recognition: base–aromatic stacking strength and the flexibility of the single-stranded nucleic acid. The model was successfully applied to predict the binding conformations of 12 distinct ssDBP and ssRBP structures with their cognate ssDNA and ssRNA partners having various sequences. Estimated binding energies agreed well with the corresponding experimental binding affinities. Bound conformations from the simulation showed a funnel-shaped binding energy distribution where the native-like conformations corresponded to the energy minima. The various ssDNA–protein and ssRNA–protein complexes differed in the balance of electrostatic and aromatic energies. The lower affinity of the ssRNA–ssRBP complexes compared with the ssDNA–ssDBP complexes stems from lower flexibility of ssRNA compared to ssDNA, which results in higher rate constants for the dissociation of the complex (*k*_*off*_) for complexes involving the former.

## Author summary

Quantifying bimolecular self-assembly is pivotal to understanding cellular function. In recent years, a large progress has been made in understanding the structure and biophysics of protein-protein interactions. Particularly, various computational tools are available for predicting these structures and to estimate their stability and the driving forces of their formation. The understating of the interactions between proteins and nucleic acids, however, is still limited, presumably due to the involvement of non-specific interactions as well as the high conformational plasticity that may demand an induced-fit mechanism. In particular, the interactions between proteins and single-stranded nucleic acids (i.e., single-stranded DNA and RNA) is very challenging due to their high flexibility. Furthermore, the interface between proteins and single-stranded nucleic acids is often chemically more heterogeneous than the interface between proteins and double-stranded DNA. In this study, we developed a coarse-grained computational model to predict the structure of complexes between proteins and single-stranded nucleic acids. The model was applied to estimate binding affinities and the estimated binding energies agreed well with the corresponding experimental binding affinities. The kinetics of association as well as the specificity of the complexes between proteins and ssDNA are different than those with ssRNA, mostly due to differences in their conformational flexibility.

## Introduction

Interactions between nucleic acids and proteins are essential and central to many biochemical processes. Protein–nucleic acid complexes have very diverse structures and the interface may depend on both the shape of the protein and the structure of the nucleic acid. The diversity of DNA and RNA sequences dictates their structures, which in turn control their binding specificity to proteins. The structure of protein–DNA complexes may vary and sometimes even small nuances in the geometrical parameters of the major or minor grooves are fundamental to achieving specificity [1,2] and therefore function. An RNA strand can fold into diverse three-dimensional (3D) structures, including double-stranded A-form helices and higher-order tertiary structures [3] that interact specifically with proteins. Stable complexes between proteins and nucleic acids are essential and their disruption can lead to a range of diseases [4], including several neurodegenerative disorders [5] and cancers [6]. Structures can be formed transiently between proteins and double-stranded DNA (dsDNA) during transcription, replication, recombination, and dsDNA repair. Structures between proteins and single-stranded (ss) DNA and RNA are also essential for function, for example, in telomeric overhangs at the end of chromosomes, at double stranded breaks, and at replication forks [7,8].

Compared with dsDNA, ssDNA structures are highly flexible [9–12] and their functional form is thermodynamically less stable, such that they are vulnerable either to forming secondary structures in which the nucleotide groups are non-accessible or to re-annealing with complementary DNA strands. They are also susceptible to detrimental chemical or enzymatic attacks. Various proteins function to specifically bind to and thereby protect ssDNA molecules so that they can take part in necessary cellular processes. Some ssDNA binding proteins (ssDBPs; often called SSBs) have the functional ability to recruit partner proteins and present the ssDNA substrate to them [13]. The structures of ssDBPs can vary in size and shape, and many of them consist of one or more copies of unique binding domains. Four such domains having distinct structural topologies have been characterized so far and their available structures reveal their mode of interaction with ssDNAs. These ssDBP domains are oligonucleotide/oligosaccharide/oligopeptide-binding (OB) folds, K homology (KH) domains, RNA recognition motifs (RRMs), and whirly domains. In a multi-domain ssDBP, domains either repeat in the same subunit or monomeric domains fold into a homo-oligomeric tertiary structure and all the domains conjointly bind ssDNA [14].

The situation is somewhat similar with respect to ssRNAs, which are an important component of RNA biology [15,16]. RNA binding proteins (RBPs) bind single-stranded RNA (ssRNA) and act either as essential cofactors for their functional activity or to protect them from degradation. The structures of ssRNA binding proteins (ssRBPs) vary in shape and size, and some of them consist of more than one copy of the binding domain. The complex structures that some of the abundant ssRBP domains form with ssRNA, such as RRMs, Pumilio repeat domains (PUFs), KH domains, OB fold domains, and tristetraprolin and CCCH-type zinc fingers (e.g., Tis11d), have been solved. However, the structural basis of their sequence specificity is often not clear.

Studying the conformational heterogeneity of ssDNA and ssRNA is challenging using common approaches because they can provide only limited information either on the global conformation or on the detailed molecular characteristics. Nevertheless, ssDNA and ssRNA were studied by atomic force microscopy (AFM; [17,18]), fluorescence resonance energy transfer (FRET;[19]), nuclear magnetic resonance (NMR;[20–22]) and small angle x-ray scattering (SAXS;[23,24]). The interactions between proteins and ssDNA or ssRNA were studied, however, the number of studied crystal structures is much smaller for ssDNA and ssRNA compared with dsDNA or dsRNA and it is unclear how they interact in solution.

Interactions between ssDNA and ssDBP or between ssRNA and ssRBP are fundamentally different from the interactions of dsDNA or dsRNA with proteins. Predicting their structures is complicated by the much greater flexibility of ssDNA/ssRNA compared with their double-stranded analogs. In many cases, ssDNA/ssRNA molecules of variable sequences but of similar length are able to adopt different conformations to engage with the same protein binding site. It was reported that the binding mode adopted is affected by salt concentration. For example, an ssDBP interacts differently with ssDNA at low and high salt concentrations [25]. In the case of ssRNA binding, although the same RRM surface is used to contact various ssRNAs, substantial variation exists in their interaction modes, in the number of interacting bases, and in their degree of specificity [26]. Additionally, the complexity of these interactions is reflected in the high thermodynamic stability of the formed complexes even when they interact with homopolymeric single stranded nucleic acids. Some of these complexes can even have an experimentally resolved structure in which the ssDNA can participate in extensive diffusion along the protein [27].

Although electrostatics (in which the negatively charged backbone of the nucleic acid is attracted to the positively charged residues on the binding surface of the protein) plays a crucial role in the interactions of both single and double-stranded nucleic acids with proteins, ssDNAs and ssRNAs are highly flexible in solution and thus they do not possess a definite shape [28]. By contrast, dsDNA and dsRNA are much more rigid and therefore their complexes with proteins often possess shape complementarity. Unlike dsDNA, the bases of ssDNA can be unstacked in the unbound form and thus are capable of engaging in π–π stacking interactions with the aromatic side chains (tryptophan (W), tyrosine (Y), phenylalanine (F), and histidine (H)) of ssDBPs. This scenario is also valid for the interaction of ssRNA with ssRBPs.

Since there is little experimental information on the conformations of ssDNA or ssRNA in solution, most reported studies have focused on the conformations of the protein. The interactions between single stranded nucleic acids and proteins have different biological functions, some of which demand sequence specificity. Complexes that are formed to protect the ssDNA from hybridization with another ssDNA are expected to be less specific and some of them were also shown to involve diffusion of the DBP along the ssDNA, so indicating the formation of various interfaces between the ssDNA and the DBP [29,30]. Indeed, several ssDBPs were crystallized with a non-specific ssDNA sequence, such as poly-T [14, 31–33]. Some ssDBP molecules, however, such as the telomere-binding proteins, bind ssDNA in a sequence-specific manner. For example, Pot1p from *S*. *pombe* binds a hexanucleotide ssDNA sequence strongly with an equilibrium dissociation constant, *K*_*D*_, in the nano-molar range but does not bind when a single nucleotide at the center of the sequence is altered [34]. For homo oligonucleotide single strand sequences (poly-A, poly-T etc.), the *K*_*D*_ equilibrium binding constant of a particular DBP can vary depending on the nucleotide type; poly-A ssDNA binds RPA with a *K*_*D*_ that is orders of magnitude higher than that of poly-T [35,36]. Even for the same binding mode, the OB-fold from cold shock protein (CSP) binds T rich sequences tighter than C rich ones [37]. Also, ssDNA sequences bind much tighter than ssRNA sequences [34, 38, 39]. In cases where the cognate ssDNA sequence binds tightly, other non-cognate sequences can also bind to the same binding site [14]. This accommodation of different sequences is possible where the protein adjusts its backbone, relocates its flexible side chains, and alters its hydrogen bonding networks and where the DNA strand undergoes structural rearrangements, mainly by rotating its bases [14,40]. Sometimes, specificity is biased toward one end of the ssDNA. For example, both *S*. *pombe* Pot1 and Cdc13 recognize a particular telomeric sequence in the 5’ region but their binding at the 3’ region is less specific [41,42].

Computational approaches can provide a powerful means of studying the complexes between proteins and ssDNA or ssRNA, particularly with respect to their dynamics and functional motions. However, only a few such studies have been reported. Atomistic molecular dynamics simulations were applied to study complexes of ssDNA with the SSB protein [43], with the RPA protein [44], and with a KH domain [45,46]. Coarse-grained molecular dynamics simulations were used to study the self-assembly of several protein-ssDNA complexes [47]. A few studies have reported the development of a computational algorithm to study the interactions of ssRNA with proteins. The major examples are an atomic distance- and orientation-based scoring function [48], a machine learning-based docking-score in RosettaDock [49], an energy-based coarse-grained force field [50,51], and a fragment-based flexible docking score [52,53]. Most of these knowledge-based algorithms were evaluated on small data sets because of the limited number of experimental structures available, which limits their coverage. Moreover, considering the RNA structure as a rigid body makes them inapplicable for the modeling of ssRNA binding, in which flexibility plays a crucial role. Applying similar approaches to ssDNA-protein complexes is challenging mostly because of the small number of available structures and low sequence similarities, which hampers efforts to construct knowledge-based potentials.

Here, we applied a physical interaction-based coarse-grained approach to construct a transferable model to study the recognition of ssDNAs and ssRNAs by ssDBPs and by ssRBPs, respectively. The method does not require any structural information on ssDNA/ssRNA, nor does it utilize any prior knowledge of the binding site. Earlier, we reported a similar model that successfully predicted the crystal complex structures of homopolymeric ssDNAs with ssDBPs coming from different domains of life [47]. New parameters have been incorporated into the current model to account for sequence-specific interactions with ssDNA/ssRNA. The two major components of the coarse-grained model that govern the interactions and stability of the complexes formed between ssDNA/ssRNA and their corresponding proteins are the flexibility of the nucleic acids and the strength of interactions between each nucleotide and the aromatic sidechains. The interface between ssDNA/ssRNA and proteins is defined by electrostatic interactions between the phosphate backbone and positively charged residues and by aromatic interactions between nucleic acid bases and aromatic residues. The sequence specificity is mostly introduced by different strengths of interactions between the four types of nucleotides (A, T/U, G, C) and the four types of aromatic side chains (W, F, Y and H). The new coarse-grained model was applied to six ssDNA–ssDBP and six ssRNA–ssRBP complexes involving binding proteins having different protein folds and ssDNA/ssRNA molecules having different lengths and sequences. The model predicted their structures successfully, was sensitive to the sequence variation of the ssDNA or the ssRNA, and qualitatively predicted their experimental binding affinities.

## Methods

### The ssDNA–ssDBPs and ssRNA–ssRBPs systems studied

For a comprehensive analysis of a variety of interactions between proteins and single-stranded nucleic acids, we studied 12 complexes: six ssDNA–ssDBP complexes and six ssRNA–ssRBP complexes whose three-dimensional structures are known (summarized in Table 1). The sets of protein–DNA and protein–RNA complexes include proteins having different functions, with folds of different sizes, and with heterogeneous ssDNA/ssRNA having different lengths and sequences. The proteins in these ssDNA–ssDBP complexes belong to different structural domains: the oligonucleotide/oligosaccharide-binding (OB) fold, the RNA recognition motif (RRM) domain, and the K homology (KH) domain. We note that the four complexes with OB-folds differ in their structures (*i*.*e*., protein length) and sequences. Likewise, the six ssRNA–ssRBP complexes were also selected to cover different structural domains, namely, the OB-fold, RRM, PUF domain, zinc-finger domain, RAMP protein, and a Fab. Overall, we covered different folds in which the electrostatic and aromatic stacking energy contributions vary from a very high stacking energy fraction (the OB-fold) to a high electrostatic energy fraction (KH-domain and RAMP). Judging from the available structures of the 12 complexes studied here and based on the available unbound structures, it appears unlikely that the proteins undergo a considerable conformational change in order to bind their ssDNA/ssRNA ligands. The ssDNA and ssRNA molecules are much more flexible in solution than folded proteins. Accordingly, one may conclude that the binding surfaces in ssDBPs and ssRBPs are predefined, and large conformational change occurs for ssDNA/ssRNA only.

**Table 1:** Studied systems of protein interactions with single-stranded nucleic acids.

	PDB ID	Protein	SSB fold/domain (#res)	DNA Seq (#nucleotide)	*λ(E_elec_ /E_arom_)
ssDNA	2ES2	Cold shockprotein from Bacillus subtilis	oligonucleotide/oligosaccharide-binding (OB) fold domain (67)	TTTTTT (6)	Very low
	2UP1	human hnRNP A1	RNA recognition motif (RRM) domain (366)	TAGGGTTAGGG (11)	0.32
	4HIO	Telomere protein Pot1pc from from S. pombe	OB-fold domain (139)	GGTAACGGT (9)	0.25
	1S40	Telomere protein Cdc13 from Saccharomyces cerevisiae	OB-fold domain (187)	GTGTGGGTGTG (11)	0.35
	1QZH	Telomere protein Pot1p from S. pombe	OB-fold domain (170)	GGTTAC (6)	0.56
	3VKE	human Poly(rC)-binding protein 1	K homology (KH) domain (79)	ACCCCA (6)	Very high
ssRNA	3PF5	Cold shock protein from Bacillus subtilis	oligonucleotide/oligosaccharide-binding (OB) fold domain (67)	UUUUUU (6)	Very low
	5E08	Synthetic antibody fragment (Fab)	Synthetic Fab for the specific recognition of ssRNA sequence (Heavy chain-234, Light chain-215)	GUAUGCAUAGGC (12)	0.20
	3V6Y	Pumilio-fem-3 mRNA binding factor 2 (PUF) from Caenorhabditis elegans	PUF domain (413)	CUGUGCCAUA (10)	0.26
	2CJK	Nuclear polyadenylation RNA binding protein 4 from Saccharomyces cerevisiae	Two RBD domains (RRM1 and RRM2) of Hrp1 (167)	UAUAUAUA (8)	0.36
	1RGO	Butyrate response factor 2 TIS11d from human	Tandem zinc finger (TZF) domain (70)	UUAUUUAUU (9)	0.65
	3QJJ	Receptor activity modifying protein (RAMP) Cas6 from Pyrococcus horikoshii	RAMP protein (239)	GUUGAAAUCAGA (12)	1.11

*λ was calculated for conformations that are similar to the crystal structures ($D_{Conf}^{1},\;D_{Conf}^{2}$ ≤ 5Å)

### Coarse-grained model for ssDNA–DBP and ssRNA–RBP

In many cases, proteins bind to cognate ssDNA or ssRNA partners in a sequence specific manner, where the binding specificity depends on the interactions between nucleotide bases and aromatic side chains. To model the sequence specific interaction of ssDNA and ssRNA with proteins, we adopted the coarse-grained model that was originally developed to study nonspecific ssDNA–ssDBP interactions [47,54].

Starting from the experimentally determined structures, the ssDBPs and ssRBPs were modeled by their native topology, where each amino acid residue was represented by two beads at the Cα- and Cβ-positions except Gly, which has only Cα. Charged amino acids were modeled by placing a point charge of +1 (Lys and Arg) or -1 (Asp and Glu) on the Cβ-bead. In some cases, His was also considered as positively charged depending on its estimated pKa values on the Propka server [55]. The ssDNA and ssRNA molecules were modeled using a coarse-grained approach as ‘beads-on-a-string’ polymers in which each nucleotide was represented by three beads representing the phosphate (P), sugar (S), and nucleo-base (B) moieties, which were positioned at the geometric center of each represented group. The phosphate bead in the model bears a -1 charge. In order to maintain chain connectivity and local geometry, the neighboring beads were constrained using bonds, bond angles, and dihedral angles. Non-bonded interactions are crucial to model the dynamics of ssDNA and ssRNA molecules. In our model, we included base-stacking interactions and hydrophobic interactions, as described below. Given the short length of ssDNA and ssRNA for all the systems studied here, the present model did not consider the possibility of intra-DNA base-pairing interactions.

### Energy function of proteins, ssDNA and ssRNA

In the simulation, the native contact interactions of the protein were maintained by the Lennard-Jones (L-J) potential, whereas nonspecific electrostatic interactions were allowed among all charged residue beads. Overall, we followed a coarse-grained protein modeling approach that was used in previous studies [47,56–58].

The internal energy of the protein *E*_*prot*_ comprises the following three bonded and three non-bonded terms:

$$E_{prot}=E_{prot}^{Bond}+E_{prot}^{Angle}+E_{prot}^{Dihedral}+E_{prot}^{Native\;contacts}+E_{prot}^{Electrostatics}+E_{prot}^{Repulsion}$$

The potential of a particular conformation Γ (Γ_0_ is the native conformation) in the molecular dynamics (MD) trajectory is then described as:

$$\begin{array}{l} E_{prot}\;(\Gamma ,\Gamma_{0})=\sum_{bonds}\;K_{bonds}(b_{ij}‐b_{ij}^{0}{)}^{2}+\sum_{angles}\;K_{angles}(\theta_{ijk}‐\theta_{ijk}^{0}{)}^{2}+\sum_{dihedrals}\;K_{dihedrals}\;[1‐\\ cos(\phi_{ijkl}‐\phi_{ijkl}^{0})-\cos(3(\phi_{ijkl}‐\phi_{ijkl}^{0}))]+\sum {}_{i\neq j}\;K_{contacts}\;[5(\frac{A_{ij}}{r_{ij}}{)}^{12}‐6\;(\frac{A_{ij}}{r_{ij}}{)}^{10}]+\sum {}_{i,j}\;K_{electrostatics}\;B(\kappa )\\ \frac{q_{i}q_{j\;exp^{-kr}}}{\varepsilon_{r}\;r_{ij}}+\sum {}_{i\neq j}\;K_{repulsion}(\frac{c_{ij}}{r_{ij}^{r}}{)}^{12} \end{array}$$

The value of the constant *K*_*bonds*_ was set to 100 kcal mol^-1^ Å^-2^, the value of *K*_*angles*_ was set to 20 kcal mol^-1^ Å^-2^ and the values of constants *K*_*dihedrals*_, *K*_*contacts*_ and *K*_*repulsion*_ were set to 1 kcal mol^-1^. For a given conformation along the trajectory, *b*_*ij*_ is the distance between bonded beads *i* and *j* and $b_{ij}^{0}$ is the optimum inter-bead distance in Å. Similarly, *θ*_*ijk*_ is the angle between sequentially bonded beads *i-k* and $\theta_{ijk}^{0}$ is their optimum angle in radians; *ϕ*_*ijkl*_ is the dihedral angle between sequentially bonded backbone beads *i-l* and $\phi_{ijkl}^{0}$ is their optimal dihedral angle in radians. Finally, *r*_*ij*_ is the distance between non-bonded beads *i* and *j* that are in contact and *A*_*ij*_ is their optimal distance in Å.

Optimal values were calculated from the atomic coordinates of the corresponding PDB structure. For the repulsion term, *C*_*ij*_ is the sum of the radii for any two non-bonded beads not forming a native contact and $r_{ij}^{r}$ is the distance between them in Å; the repulsion radii for the backbone and side chain (Cβ) beads were set to 1.9 Å and 1.5 Å, respectively. The electrostatic interactions were modeled by the Debye-Hückel potential, and we followed the parameters used in previous studies in our group [57,58]. In the coarse-grained model, the inherent flexibility of protein segments varies as a function of the density of the native contacts in the local surroundings. In addition, we incorporated enhanced flexibility for segments either with high B factors (*i*.*e*., higher than the mean B factor) or with missing coordinates. For complexes that were resolved by NMR (1s40.pdb), the flexible regions were predicted using FlexServ [59]. In order to retain the unimpaired native fold of the protein including its binding site, all simulations were run at relatively low temperatures to allow the protein to fluctuate around its native state but not to unfold.

All simulations were started from the extended conformation of the ssDNA or ssRNA. In contrast to the modeled proteins, there were no native contact interactions for ssDNA/ssRNA. There are several models for ssDNA that aim to capture sequence-dependent polymeric properties (e.g., persistence length and force-extension profiles) [60–65]. The current model was based on one we developed for homopolymeric ssDNA that successfully predicted binding with ssDBPs[47]. In this model, intra-molecular electrostatic repulsions were not allowed between negatively charged phosphate beads, and the ssDNA/ssRNA flexibility was modulated by the two dihedral potentials described below. Consistently with previously reported studies [50,60,61,66], the following are the potential energy terms of the ssDNA and ssRNA used in our model:

$$E_{ssDNA/ssRNA}=E_{ssD/RNA}^{Bond}+E_{ssD/RNA}^{Angle}+E_{ssD/RNA}^{Dihedral}+E_{ssD/RNA}^{Base\;pairing}+E_{ssD/RNA}^{Stacking}+E_{ssD/RNA}^{Repulsion}$$

Here, the first three terms are responsible for retaining the ssDNA/ssRNA backbone and their forms are identical to the corresponding terms in *E*_*prot*_. The term $E_{ssD/RNA}^{Bond}$ represents the contribution from the covalently linked beads and comes from the following bead-pairs: (*P*_*i*_*-S*_*i*_), (*S*_*i*_*-B*_*i*_), and (*S*_*i*_*-P*_*i+1*_) with *K*_*bonds*_ = 100 kcal mol^-1^ Å^-2^. The term $E_{ssD/RNA}^{Angle}$ is the bond angel potential and comes from the following bead-trios: (*P*_*i*_*-S*_*i*_*-B*_*i*_), (*B*_*i*_*-S*_*i*_*-P*_*i+1*_), (*P*_*i*_*-S*_*i*_*-P*_*i+1*_), and (*S*_*i*_*-P*_*i+1*_*-S*_*i+1*_), with *K*_*bonds*_ = 20 kcal mol^-1^ Å^-2^. The term $E_{ssD/RNA}^{Dihedral}$ is the potential for the dihedral angles included to mimic the flexibility of ssDNA or ssRNA in the model. We introduced two types of dihedral potentials: i) those formed between the following four consecutive base and sugar beads to modulate the flexibility of the base–sugar moiety: *B*_*i*_, *S*_*i*_, *S*_*i+1*_, and *B*_*i+1*_ with *K*_*dihedrals*_ = 0.5 kcal mol^-1^ and 1.5 kcal mol^-1^ for ssDNA and ssRNA, respectively; and ii) those formed between four consecutive phosphate beads to modulate the flexibility of the phosphate backbone: with *P*_*i*_, *P*_*i+1*_, *P*_*i+2*_, and *P*_*i+3*_ *K*_*dihedrals*_ = 0.3 kcal mol^-1^ and 0.9 kcal mol^-1^ for ssDNA and ssRNA, respectively. The values were calibrated so that the persistence length of ssDNA/ssRNA calculated from the simulations qualitatively resembled that observed in experiments (see below). The values of the native bond lengths and angles were obtained from the PDB atomic coordinates of the helical structure that ssDNA adopts in the duplex form.

The first two terms in the potential energy equation dictate the connectivity of the ssDNA/ssRNA and the other four terms dictate the global conformation. Base-pairing and base stacking may contribute to the structural stability of the ssDNA/ssRNA. All ssDNA and ssRNA systems studied here were of short length, moreover, the homopolymeric nature of some of the sequences restricted the possibility of base-pair formation. We thus set $E_{ssDNA}^{Base\;pairing}$ = 0 and kept this potential for future studies with longer ssDNA segments.

The attractive nature of the π-stacking between consecutive bases was incorporated by using a short range L-J potential between consecutive ssDNA/ssRNA bases: $E_{ssDNA}^{Stacking}=‐\varepsilon_{B-B}[5(\frac{r_{ij}^{0}}{r_{ij}}{)}^{12}‐6(\frac{r_{ij}^{0}}{r_{ij}}{)}^{10}]$, with $r_{ij}^{0}$ being the typical distance between consecutive bases and set to 3.6 Å[67]. Different stacking interaction strengths (*ε*_*B*−*B*_) represent different depths of the potential well between the neighboring stacked bases and can take different values depending on the nature of the two bases. Previous efforts have endeavored to estimate the interaction energies of stacked nucleobase dimers experimentally [68] and from quantum chemical calculations [69]. Though obtained differently, their trends are similar, as expected. For example, in both cases, Guanine was found to have lower interaction energies (engage in stronger interactions) compared with Thymine. This set of interaction energies was used to assess base–base stacking interaction strengths in ssDNA coarse-grained models to elucidate ssDNA dynamics [70] and DNA hybridization[71]. We adopted the energetic values for stacking *ε*_*B-B*_ for different base pairs from an earlier study [71] and rescaled the values to fit the experimental persistence length of poly-T ssDNA (Table 2). In the model, the base stacking is strongest for purines and weakest for pyrimidines. Adopting an approach similar to that used with the proteins, we applied a repulsion term (*i*.*e*., excluded volume) to all non-bonded beads in ssDNA/ssRNA. This repulsion energy was applied to any beads of non-adjacent nucleotides; the radii of the base, phosphate and sugar beads were 1.5 Å, 3.7 Å, and 3.7 Å, respectively.

**Table 2:** Base-base stacking interaction strengths.

Base stack pair	Energy (kcal/mol)	Base stack pair	Energy (kcal/mol)
AA	1.4	AC	1.5
TT	1.0	TG	1.7
GG	1.8	TC	1.4
CC	1.4	GC	1.5
UU	0.9	AU	1.3
AT	1.4	GU	1.5
AG	1.8	CU	1.3

### Modeling the flexibility of ssDNA and ssRNA

A major challenge in predicting the complexes formed between proteins and ssDNA/ssRNA stems from the considerable flexibility of the latter. Their flexibility is linked to electrostatic repulsions between negatively charged phosphate groups and can, therefore, be modulated by salt concentration. Indeed, the persistence length of ssDNA decreases whereas its contour length increases with increasing salt concentration [12,19,72]. In our coarse-grained simulation, the effect of salt concentration was incorporated by using the Debye-Hückel potential, which modulates the ssDNA persistence length as well as electrostatic interactions at the protein–ssDNA/ssRNA interface.

To modulate the flexibility of the ssDNA and ssRNA, we omitted ion condensation effects and simplified the representation of the effect of electrostatics on the ssDNA/ssRNA persistence length by adding the two dihedral potentials described above. We calculated the persistence length (*L*_*ps*_) of the modeled ssDNA and ssRNA using the expression for a flexible polymer (*L*/*L*_*ps*_ >> 1): *L*_*ps*_ = <*Ll*_*0*_>/<*l*_*0*_>, where *l*_*0*_ is the vector between the first two monomers (the bond vector between the two phosphate beads at the 5’ end), and *L* is the end to end vector (the bond vector between the phosphate beads at the 5’ and 3’ end) of the polymer. In the model, *L*_*ps*_ for a T_40_ polymer initially increased with the backbone dihedral potential (from 0 to 1.2 kcal mol^-1^) and saturated thereafter. The persistence length obtained using this approach is in agreement with experimental values and is consistent with the values from other computational approaches[47].

Here, the values of K_dihedral_ were chosen such that the relative persistence lengths of ssDNA and ssRNA agreed with the experimentally determined range. The experimentally reported values of the persistence lengths of ssDNA and ssRNA span a wide range of 1.0–6.0 nm that is sensitive to the solution condition (e.g., ionic strength and ion types) and experimental technique (e.g., FRET, SAXS, and AFM). Several studies reported higher persistence length for ssRNA than ssDNA [12,19]. In a recent comparative study, by fitting SAXS data with a worm-like chain model, the persistence length of dT_40_ (16–19 Å) was found to be less than that of dU_40_ (19–22 Å) at a particular salt concentration [12]. We mimicked the lower persistence length of ssDNA by modeling it with a lower backbone dihedral constant (K_dihedral_ = 0.3) and the higher persistence length of ssRNA with a higher dihedral constant (K_dihedral_ = 0.9). The *L*_*ps*_ values for ssDNA and ssRNA were 30 Å and 42 Å, respectively, which is in the range of the experimentally measured flexibility of ssDNA and ssRNA [11,67]. These values yielded a persistence length for ssRNA that was 30% larger than that of ssDNA, consistently with the ratio estimated by the SAXS measurements [12]. This difference between the flexibility of ssDNA and ssRNA was needed to reproduce their different binding affinities to ssDBPs and ssRBPs, respectively.

### Protein–ssDNA/ssRNA interaction energy function

In our model, the interaction potential between a protein and ssDNA/ssRNA comprises the following three components: (i) the electrostatic interaction between the Cβ-beads representing the side chain of charged residues (K, R, H, D, and E) and the negatively charged phosphate beads of ssDNA/ssRNA; (ii) the aromatic stacking interaction between the Cβ-beads representing aromatic side chains (W, F, Y, and H) and the ssDNA/ssRNA base bead; and (iii) the repulsive interactions between other beads of the protein and ssDNA/ssRNA. Thus,

$$E_{prot-ssD/RNA}=E_{prot-ssD/RNA}^{Electrostatics}+E_{prot-ssD/RNA}^{Aromatic}+E_{prot-ssD/RNA}^{Repulsion}$$

The electrostatic interactions between all of the charged beads in the system are modeled by the Debye–Hückel potential. These interactions are nonspecific, and the phosphate groups of the ssDNA/ssRNA can interact with any charged residue of the ssDBP or ssRBP, respectively. The repulsion is applied to all beads of the protein and all beads of the ssDNA/ssRNA.

**Fig 1:**
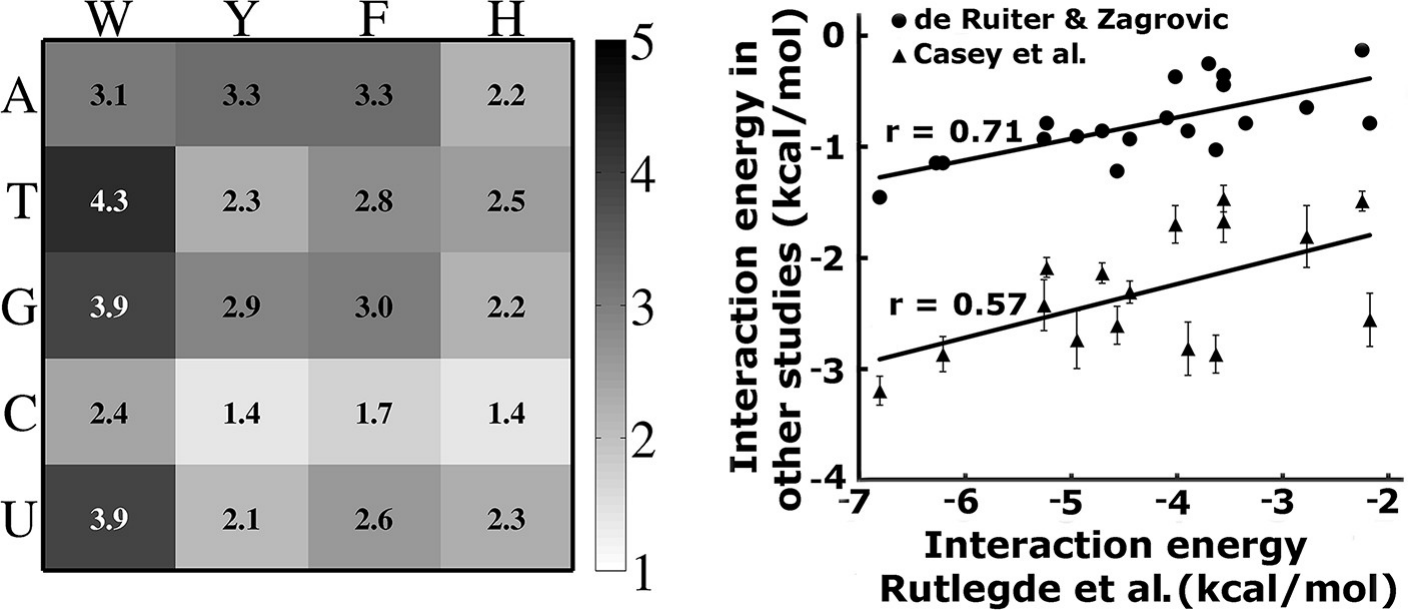
The strength of the specific stacking interactions between different nucleobase–aromatic Cβ bead pairs (*ε*_*B*−*AA*_). A). Parameters were derived from solvent-phase quantum calculations of base–aromatic stacked dimers (reported by Rutledge et al.,[73]) and rescaled according to the coarse-grained model. B). Interaction energies derived from Rutledge et al., are compared with values from nucleotide-residues from de Ruiter et al., [75]and Andrew et al.,[76].

Unlike dsDNA, the nucleobases of extended ssDNA/ssRNA are free to engage with aromatic residues in π–π stacking, which plays a crucial role in protein–ssDNA/ssRNA interactions. These stacking interactions were characterized and compared using detailed quantum chemical calculations [73,74]. The energies of these interactions were estimated to range between -9.4 and -28.5 kJ∙mol^-1^ in water; suggesting that the π–π stacking interactions play an important role in stabilizing the interface between proteins and ssDNA/ssRNA. Stacking energy increases with the amino acid according to Phe < His ≈ Tyr < Trp, while the stacking energy is generally larger for purines compared with pyrimidines[73]. Similarly to base stacking, the aromatic interactions between the Cβ-beads of aromatic side chains (W, F, Y, and H) and the nucleotide base bead is also modeled by the L-J potential and weighted by the base–aromatic interaction strength *ε*_*B*−*AA*_ (Fig 1). Thus,

$$E_{ssDNA/ssRNA}^{Aromatic}=‐\varepsilon_{B-AA}\;[5(\frac{r_{ij}^{0}}{r_{ij}}{)}^{12}‐6(\frac{r_{ij}^{0}}{r_{ij}}{)}^{10}]$$

where $r_{ij}^{0}$ is the average distance between an aromatic side chain and a base and was set as 3.6 Å. The value of *ε*_*B*−*AA*_ varies depending on the *B-AA* pair. We adopted these pairwise base-aromatic energy values from the studies of Rutledge et al.,[73]. To scale these values to fit appropriately into our model, we reweighted the sets of *ε*_*B*−*AA*_ values by a factor of 0.15 to maximize the populations of the native state binding mode and to minimize its binding energy.

**Fig 2:**
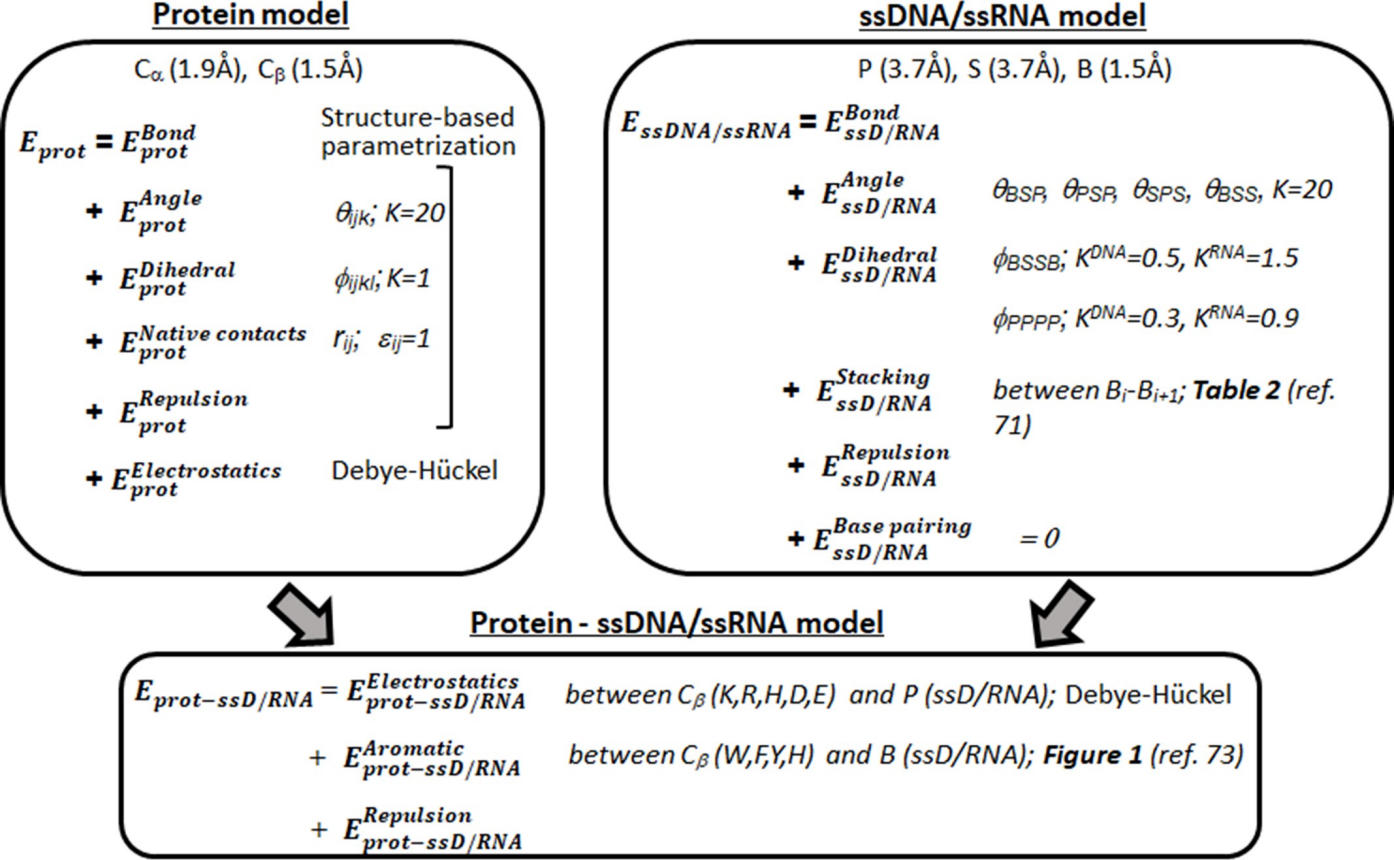
A scheme of the major components of the model for protein interactions with single-stranded nucleic acids.

The composition of ssRNA differs from that of ssDNA only by a single nucleotide (uracil in place of thymine). Based on their chemical similarity, uracil and thymine are expected to have similar stacking energies. Indeed, it was estimated that the stacking energy for uracil is only 8–10% lower than that of thymine[73,74]. In the model, the base–aromatic energy for ssDNA and ssRNA only differs for uracil and thymine. We compared the *ε*_*B*−*AA*_ values used in the model with the similar values reported recently, which were calculated by the potential mean force [75] and free energy estimation [76] methods from all-atom molecular dynamics simulations with explicit water. The *ε*_*B*−*AA*_ parameters in the model correlated well with both of these sets, the corresponding correlation coefficients are 0.57 and 0.71 with the potential mean force and free energy methods, respectively (Fig 1B). We note that the correlation coefficients improved when the values corresponding to interactions with Tyr were excluded (r = 0.80 and 0.87, respectively). The details of the model are schematically summarized in Fig 2.

### Conformational sampling and analysis

The dynamics of the protein and ssDNA were simulated using Langevin dynamics and deploying the total potential energy ***E***_***prot***_ + ***E***_***ssDNA*/*ssRNA***_ + ***E***_***prot***−***ssD***/***RNA***_ of the system. All simulations were performed with an implicit solvent model of dielectric constant 70 (water) and at a 10 mM salt concentration. We point out that, because of the coarse-grained representation of the systems, the effective salt concentration may correspond to a higher value (by a factor of ~3) than for an atomistic representation. We chose a temperature of 0.3 (arbitrary units), at which the protein was shown to fluctuate around its native fold, and the ssDNA/ssRNA was able to perform an extensive search that included diffusion over the protein surface. At this temperature, the bound state was thermodynamically more favorable and thus more populated than the dissociated state. Importantly, at this temperature, the persistence length of the modeled ssDNA/ssRNA fit the related experimental values.

The model was initially tested for its ability to maintain the native bound structure of all of the six ssDNA–DBP complexes and the six ssRNA–RBP complexes when the simulations were started from the bound conformations. We started the predictive simulations by placing an unbound ssDNA/ssRNA molecule (in its linear form) at one of six different positions around the DBP or RBP at a distance of 35–40Å. For each ssDNA/RNA position, 100 replications were performed and, in each case, a unique random seed was used to generate different velocity distributions. Thus, a total of 600 simulations were run for each system in order to perform extensive sampling of association mechanisms having multiple binding routes. Each trajectory was simulated for 10^7^ molecular dynamics steps with a time step of 0.005. Conformations were saved every 1000 molecular dynamics steps, thus 10000 conformations were saved in each trajectory. Finally, to consider the part of the trajectory that was equilibrated well, the last 2000 conformations were collected from each trajectory for analysis, such that we analyzed a total of 12 × 10^5^ (6 × 100 × 2000) conformations per system.

To evaluate the sampled conformational ensemble and especially to examine the deviation of the simulated bound conformations from the experimental structure, we utilized two similarity parameters: *D*_*Site*_ and *D*_*Conf*_. Both these parameters quantify the similarity between the crystal and simulated conformations of the binding interface in the ssDNA–protein or ssRNA–protein complex. The *D*_*Site*_ term achieves this by probing the protein patch used for interaction with either ssDNA or ssRNA, whereas the *D*_*conf*_ term probes the conformation of the ssDNA/ssRNA in this site. Consequently, *D*_*Conf*_ is sensitive to whether the ssDNA/ssRNA is located at the binding site from 3' to 5' or vice versa, however, *D*_*Site*_ quantifies the location and conformation irrespective of ssDNA/ssRNA directionality. To calculate *D*_*Site*_ and *D*_*Conf*_, the Cβ-beads of all the positively charged (K and R) as well as the aromatic (W, Y, F and H) residues in the experimentally resolved structures of the ssDNA–DBP or ssRNA–RBP interfaces were identified. Any positive residue bead lying within a cutoff distance (9 Å) from any phosphate bead, and any aromatic residue bead lying within the same cutoff distance from any base bead were defined as the native interfacial residues. The size of the interface varied between the different systems.

The following equation was then applied to the interfacial residue and the ssDNA/ssRNA phosphate or base to calculate the crystal structure similarity parameter, *D*_*Site*_.

$$D_{Site}=\frac{1}{N_{prot}\;N_{ssDNA}}\sum_{i}^{N_{prot}}\left(\sum_{j}^{ssDNA}r_{ij}-\sum_{j}^{ssDNA}r_{ij}^{0}\right)$$

Here, *i* and *j* are the i^th^ bead of the selected Cβ of the protein and the j^th^ bead of the ssDNA/ssRNA, respectively, and thus, r_ij_ and r_ij_^0^ are the pairwise distances between those beads in the simulated structure and the crystal structure, respectively. The pairwise distances were calculated either between each selected aromatic amino acid and all of the base beads of the ssDNA/ssRNA or between each of the selected charged amino acids and all of the phosphate beads of the ssDNA/ssRNA. *N*_*prot*_ is the total number of selected interfacial amino acid residues (positively charged or aromatic), and *N*_*ssDNA*_ is the number of nucleotides in the length of ssDNA/ssRNA examined. Thus, the term *D*_*Site*_ quantifies the overall conformational similarity between the predicted binding interface in the ssDNA–protein complex and the crystal structure, with a *D*_*Site*_ = 0 indicating 100% conformational similarity.

To obtain a finer structural quantification of the interface, we divided the selected interfacial residues into two groups that covered two different regions of the interface. We then calculated two order parameters, *D*^*1*^_*Site*_ and *D*^*2*^_*Site*_, each characterizing the accuracy of the prediction for the corresponding region of the interface. The advantage of the *D*_*Site*_ measure (compared with other structural measures, such as root mean square deviation (RMSD)) is that it quantifies the location and conformation of the ssDNA/ssRNA relative to each potential interfacial residue of the ssDBP or the ssRBP and ignores the directionality of the ssDNA/ssRNA. For example, a situation in which the ssDNA (which is poly T and lacks polarity in the model) is perfectly located at the protein interface but is flipped from 3′ to 5′ (instead 5′ to 3′) will result in a large RMSD value but a very low *D*_*Site*_ value.

The other structural similarity parameter, *D*_*conf*_, was calculated where the 5’ to 3’ direction of the bound ssDNA/ssRNA was taken into consideration. The same set of interface residues was identified first using the same criteria as used for *D*_*Site*_. Next, a list was made of native pairwise interactions between a base bead and the Cβ-bead of the nearest aromatic residue or between a phosphate bead and the Cβ-bead of the nearest positively charged residue. The following equation was then applied to this pairwise interfacial interaction to calculate *D*_*conf*_:

$$D_{conf}=\frac{1}{N_{ssDNA}}\sum_{i}^{N_{ssDNA}}\left(r_{i}-r_{i}^{0}\right)$$

Where, *r*_*i*_^0^ is the distance of the *i*^*th*^ pair of base–aromatic or phosphate–positive beads from the above list, *r*_*i*_ is the corresponding value for the simulated structure. *N*_*ssDNA*_ is the total number of ssDNA/ssRNA base and phosphate beads. Thus, the term *D*_*conf*_ quantifies the conformational similarity between the predicted binding interface in the ssDNA/ssRNA–protein complex and the crystal structure considering ssDNA/ssRNA direction. As with *D*_*Site*_, *D*_*conf*_ = 0 Å corresponds to 100% conformational similarity.

The values of *D*_*Site*_ and *D*_*Conf*_ can be calculated also for a specific region of the interface formed between the ssDNA or the ssRNA and the protein. In this case, for *D*_*Site*_ only the relevant interfacial residues will be used and for *D*_*Conf*_ the relevant pairwise distances will be taken into account.

## Results

### Structural classification of the ssDBP and ssRPB folds studied

Although different ssDBPs and ssRBPs perform different cellular functions, the actual number of distinct domains found in both cases is limited. The ssDBPs arrange these domains in a modular way to achieve different structures for distinct activities, including ligand specificities. In this study, we considered all three types of ssDBP domain whose complete structures are available, namely, OB folds, KH domains, and RRMs. We studied four different kinds of single OB-fold structures of variable sizes (67 to 187 residues) and different lengths and sequences of ssDNA (Table 1). These proteins are capable of binding specific ssDNA sequences with different affinities (particularly for the telomere proteins). The six studied ssDNA–ssDBP complexes differ in the relative contributions made by electrostatic and aromatic energies. For example, ssDNA typically binds OB-folds such that the bases facing the protein participate in both intra- and inter-molecular aromatic stacking interactions and the backbone remains exposed to the solvent, but for the KH domain, the electrostatic energy is much larger. For simplicity, we did not include multi-domain ssDBPs, such as PARP1 [77], which bind folded ssDNA with definite secondary structures, or proteins such as RPA, which demand high flexibility (*i*.*e*., undergo conformational changes) in order to bind ssDNA [33].

The selected ssRNA–ssRBP systems (Table 1) represent the four most abundant ssRNA-binding domains in proteins: RRMs, PUF, CCCH-type zinc fingers, and OB fold domains. Their abundance suggests that these folds have the versatility to function as diverse recognition modules. Indeed, they possess modular structures of multiple repeats that arrange to create versatile RNA-binding surfaces. Additionally, two more unique structures, an engineered synthetic antibody fragment and a RAMP that binds single-stranded CRISPR Repeat RNA, were also included. The RRM is among the most abundant structural motifs and approximately 500 human proteins contain RRMs, often in multiple copies in the same polypeptide chain.

### Prediction of binding modes of ssDNA/ssRNA-protein interactions

The performance of the developed coarse-grained model in studying protein–ssDNA/ssRNA interactions was tested by quantifying the binding mode of the sampled conformations of the twelve simulated systems and by comparing them with the corresponding X-ray or NMR structures. Considering both the flexible nature of ssDNAs/ssRNAs and their linear shape, a more detailed structural comparison can be achieved by dividing the ssDNA–ssDBP and the ssRNA–ssRBP interfaces of the experimental structures into two moieties. Splitting the interface into two moieties is useful to estimate the similarity of each of them to the corresponding region in the experimentally resolved structure. Thus, in the context of calculations to determine the similarity between the crystal and simulated conformations of the binding interface in the ssDNA–protein or ssRNA–protein complex, *D*^*1*^_*Site*_ and *D*^*2*^_*Site*_ indicate whether the ssDNA or ssRNA interacts with the native patches on the protein linked with the two moieties that comprise the experimental interface. Similarly, *D*^*1*^_*Conf*_ and *D*^*2*^_*Conf*_ describe the conformation and directionality of the ssDNA or ssRNA (details in Methods). We note that the two moieties have similar contribution to the interface stability (each contributes 40–60% to the interface energy). *D*_*Conf*_ thus reflects the molecular identity of the interactions at the interface and is a more appropriate measure than *D*_*Site*_ when examining binding specificity. Small values for these structural measures correspond to conformations having a greater degree of similarity with the experimental structure, in which ($D_{Site}^{1},\;D_{Site}^{2}$) or ($D_{Conf}^{1},\;D_{Conf}^{2}$) equals (0, 0).

**Fig 3:**
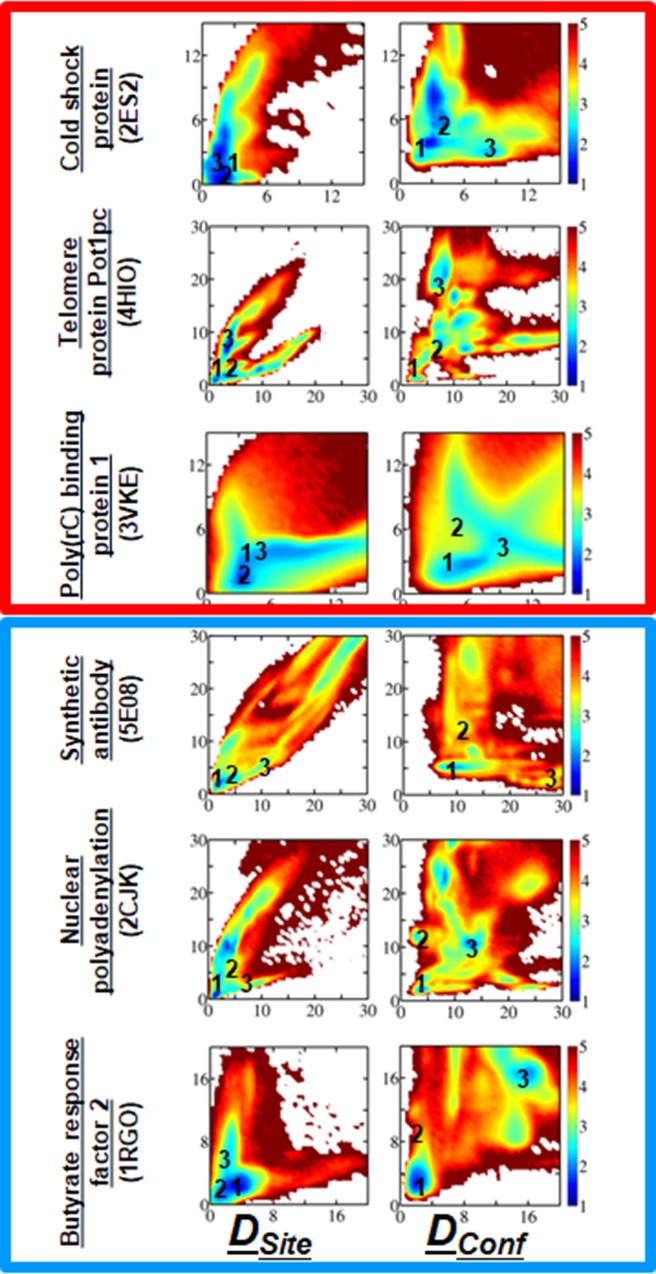
Conformational ensemble of predicted structures of proteins with single-stranded nucleic acids. The population distribution of predicted conformations is shown for ssDBP–ssDNA (top, red square) and ssRBP–ssRNA (bottom, blue square) complexes. The simulated structures are quantified by two similarity parameters, *D*_*Site*_ and *D*_*Conf*_. Whereas *D*_*Conf*_ quantifies the conformational similarity between the predicted and experimental interface considering ssDNA/ssRNA direction, *D*_*Site*_ quantifies their overall conformational similarity without considering ssDNA/ssRNA direction. Accordingly, *D*_*site*_ mostly highlights the accuracy of the predicted binding patch of the protein and *D*_*conf*_ also sheds light on the specificity of interactions at the interface between the protein and the ssDNA/ssRNA (see Methods). To rank each predicted conformation by its similarity to the experimentally determined structure, the interface was divided into two moieties (1 and 2) and the deviation of each moiety from the corresponding region of the experimental structure was measured. A lower value of *D*_*conf*_ (or of *D*_*site*_) corresponds to a conformation having a greater degree of similarity to the experimental structure, whose *D*_*Site*_ values (*D*^*1*^_*Site*_, *D*^*2*^_*Site*_) and *D*_*Conf*_ values (*D*^*1*^_*Conf*_, *D*^*2*^_*Conf*_) are (0,0). The colour bar shows the free energy of the different binding conformations of the complex, where the scale ranges from blue (low energy, densely populated) to red (high energy, sparsely populated). Representative conformations from three regions marked 1–3 in the current figure are shown in Fig 4. Additional molecular and structural details for each of the complexes can be found in Table 1.

**Fig 4:**
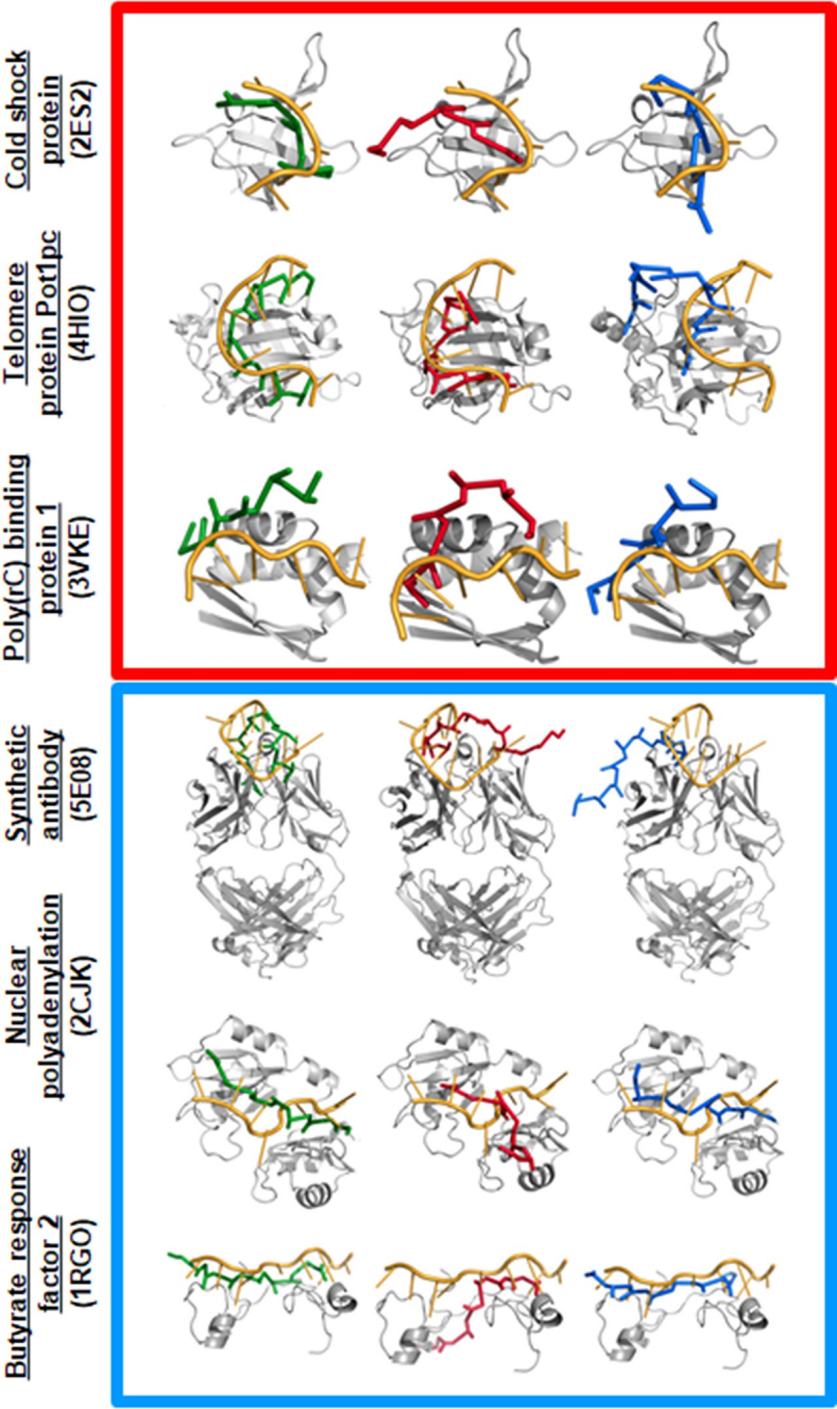
Three representative conformations for ssDBP-ssDNA and ssRBP-ssRNA. The conformations are sampled from simulations that correspond to the densely populated regions labelled 1, 2, and 3 in Fig 3 are shown in green, red, and blue, respectively, for each of the ssDBP–ssDNA (top, red square) and ssRBP–ssRNA (bottom, blue square) complexes. All-atom cartoon representations of the protein (in gray) and of the bound conformation of the ssDNA or ssRNA (in orange) are shown for comparison. The lowest energy green ssDNA/ssRNA conformations (region 1) are most similar to the orange experimental conformations (lower values of $D_{Conf}^{1}$ and $D_{Conf}^{2}$ and of $D_{Site}^{1}$ and $D_{Site}^{2}$), which demonstrates the predictive power of the model.

Fig 3 shows the sampled conformational ensembles for three simulated ssDNA–ssDBP complexes and three ssRNA–ssRBP complexes projected along ($D_{Site}^{1},\;D_{Site}^{2}$) or ($D_{Conf}^{1},\;D_{Conf}^{2}$) (the other six simulated ssDNA–DBP and ssRNA–RBP conformations are shown in the Supporting Information). The free energy surface of the binding process for each of the different studied folds (in which the interaction between the ssDBP and ssDNA or between the ssRBP and ssRNA was modeled by combining electrostatic and aromatic interactions) reflects that, in all six cases, near-native conformations with low values of $D_{Site}^{1}$ and $D_{Site}^{2}$ are highly populated (blue in Figs 3 and S1). We note that the sequence independent model captures the complexes of telomeric proteins with polyT ssDNA [47] similarly to that using the sequence-dependent model, yet with lower probabilities (S2 Fig). Three representative conformations of each of the studied complexes that correspond to densely populated (*i*.*e*., low total energy) regions are shown in Figs 4 and S3 for the six systems. We note that in all cases, the ssDNA and ssRNA conformations possessing minimum binding energies bind at or very close to the actual binding site.

A more heterogeneous conformational space is illustrated when projecting the sampled structures along ($D_{Conf}^{1},\;D_{Conf}^{2}$), which measures not only the conformation of the DNA at the binding site but also its directionality (*i*.*e*., 5’ to 3’, see Methods). These maps show that near-native conformations with low values of $D_{Conf}^{1}$ and $D_{Conf}^{2}$ are reasonably populated. Decomposition the contribution of the ssDNA/ssRNA backbone and bases to the accuracy of the predicted near native conformations (region 1), reveal that the accuracy of the backbone conformation is slightly higher by about 2Å than the predicted conformations of the bases (S4 Fig). A few additional regions, however, corresponding to non-native conformations of the DNA, are also found to be populated, and some of them possess low binding energy. Their representative conformations in Fig 4 suggest that, although they bind to the actual binding site with a similar alignment but a different orientation to that of the experimental structure (the 5’ and 3’ ends are flipped), their binding energy approaches the minimum. Overall, similar trends were found for the six remaining systems (see Supporting Information).

### Funneled energy landscape for binding in ssDBP–ssDNA and ssRBP–ssRNA complexes

**Fig 5:**
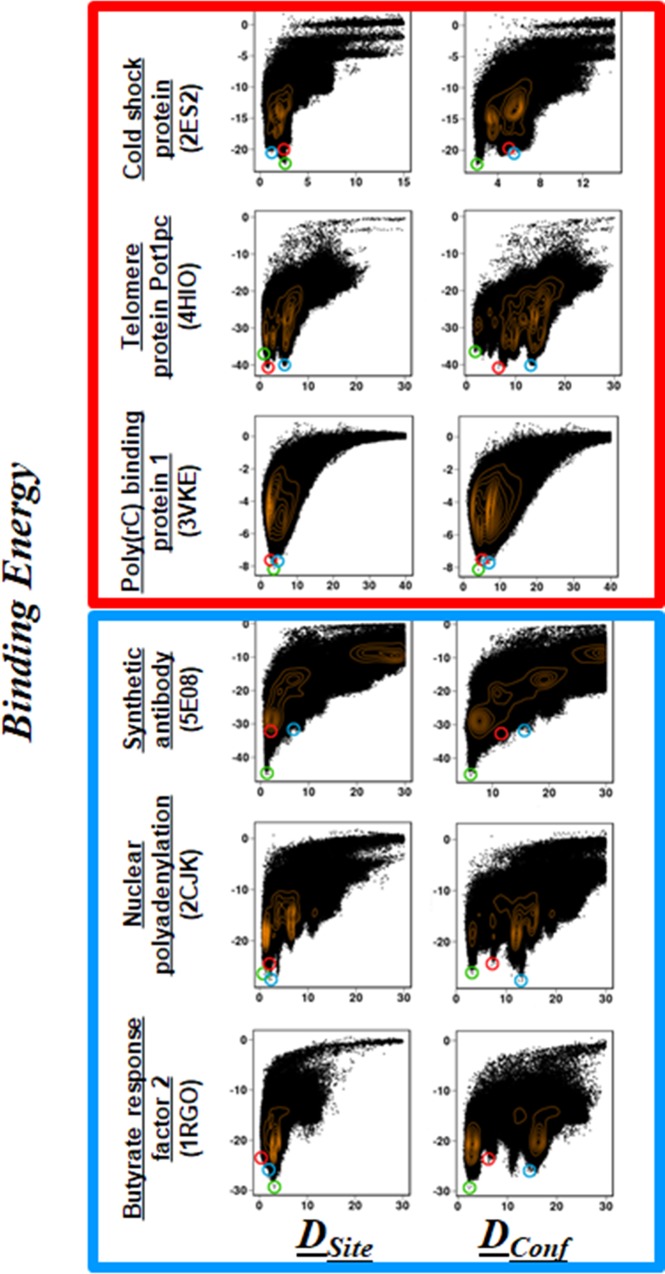
Energy landscapes for simulated ssDNA–ssDBP and ssRNA–ssRBP complexes. The binding energy (Kcal mol^-1^) is plotted versus *D*_*Site*_ and *D*_*Conf*_ for each of the ssDBP–ssDNA (top, red square) and ssRBP–ssRNA (bottom, blue square) complexes. The points encircled in green, red, and blue correspond to the respective ssDNA/ssRNA conformations shown in Fig 4. The population density of the ssDNA/ssRNA ensemble is shown by orange contour lines. A funnel-shaped binding energy landscape is present in all cases, with ssDNA/ssRNA conformations closest to the experimental structures possessing the minimal energy.

To examine the shape of the binding energy landscape for the interaction of proteins with single stranded nucleic acids, we plotted the potential energy of binding, E_bind_ (*i*.*e*., E_ssDNA/ssRNA-Prot_), for the simulated systems along *D*_*Site*_ or *D*_*Conf*_ (Fig 5). For all 12 systems, the distribution of *D*_*site*_ follows a funneled energy landscape in which near-native structures correspond to a lower binding energy. When the direction of the DNA is not considered in the structural measure, the distribution shows a more funneled shape, with the near-native structures at the minimum energy positions for all proteins except for the two sequence-specific telomere proteins Pot1pc (4HIO, Fig 5) and Cdc13 (1S40, S5 Fig), which have a rugged bottom in their binding free energy surface. Indeed, Pot1pc can accommodate ssDNAs with variable sequences by adjusting the side chains of its interface residues[78], whereas Cdc13 shows variable specificity at the two terminals of the ssDNA[42]. Similarly, for the ssRNA–ssRBP complexes, plotting the binding energy along *D*_*Site*_ reveals global funneled energy landscapes. However, when the order parameter is described by *D*_*Conf*_, a more rugged landscape is observed for some systems (e.g., Pot1pc and nuclear polyadenylation). This suggests that the detailed conformations of ssDNA and of ssRNA at more rugged binding sites can vary, and their energies can compete with that of the native conformation of the single-stranded nucleic acids.

### Role of nucleotide sequences in binding

Various factors may affect the specificity of the recognition between proteins and nucleic acids. Major determinants for specificity are the conformational ensembles of the two molecules in solution and the network of interactions (e.g., aromatic, charged–charged interactions, and hydrogen bonds) at the interfaces between the proteins and the nucleic acids [14,40,79]. In our model, sequence specificity is expected to be governed by aromatic–base interactions rather than by electrostatic interactions. We note that, in eleven of the twelve systems studied here, >30% of the total aromatic side chains are located at the binding interface. The only exception is the KH domain, which uses solely electrostatic interaction. We postulate that stacking interactions between specific ssDNA or ssRNA bases and aromatic side chains play a major role in sequence-specific binding.

**Fig 6:**
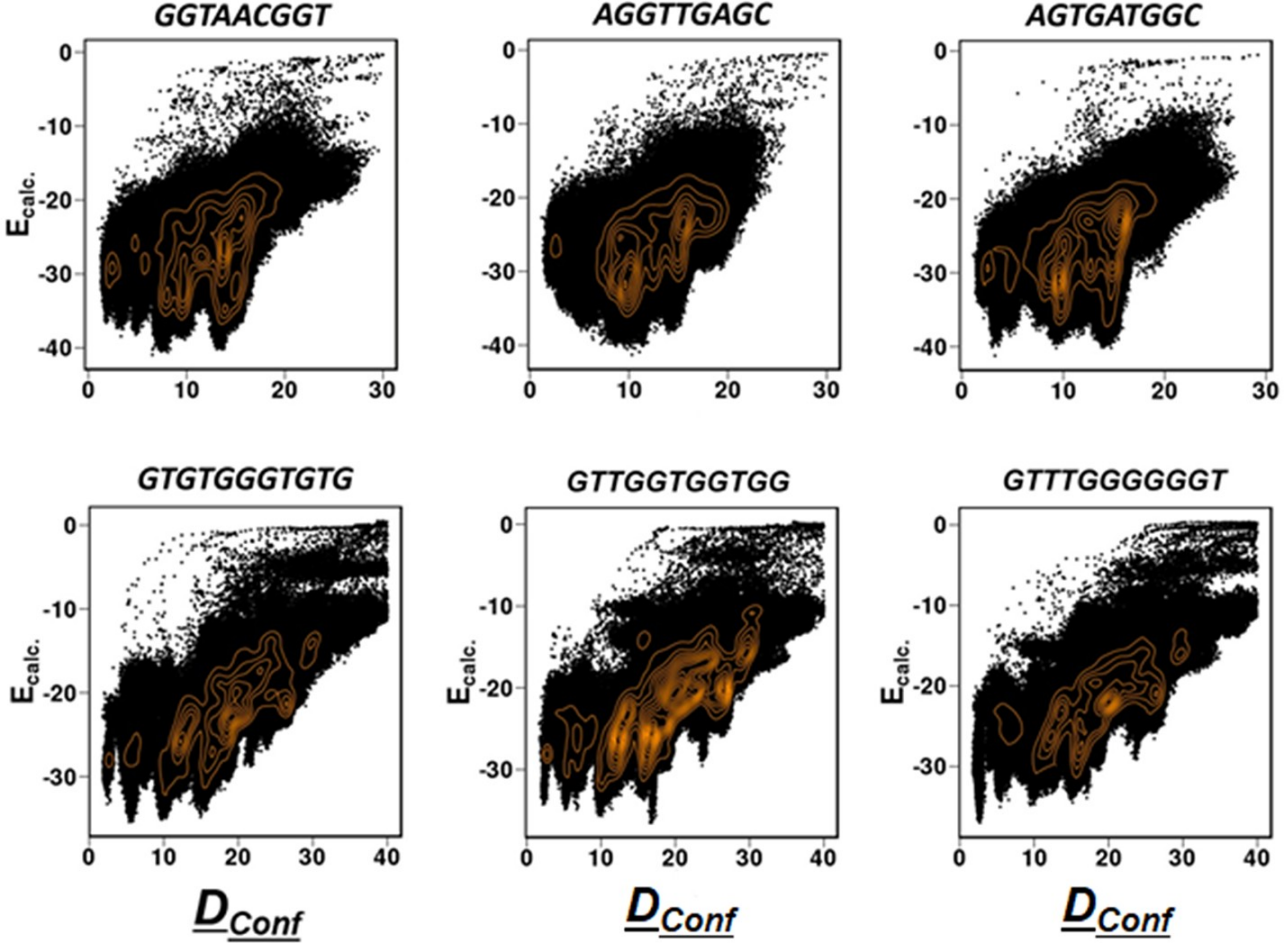
Plots of *D*_*Conf*_ *vs*. the calculated binding energy. The sequence variants of two DBP–ssDNA complexes: Pot1pc (4HIO.pdb, top line) and Cdc13 (1S40.pdb, bottom line). Two representative sequence variants, one with better (right column) and the other with inferior (middle column) binding specificity with respect to the wild type sequence (left column) are shown.

We examined the degree of specificity by investigating the effect that shuffling of the nucleic acid sequences had on the binding energy landscape with the corresponding protein. For this, we chose two telomeric proteins that are expected to interact specifically with ssDNA. We note that some ssDBPs interact similarly with homopolymeric ssDNA (e.g., polyT) and thus are not sensitive to ssDNA sequence. For each telomeric ssDNA sequence, a few other sequences were designed by shuffling the nucleotides while keeping their content fixed and then examining whether the binding pattern changed in the shuffled sequences. The energy landscape for the shuffled sequences (depicted by plotting E_bind_ (*i*.*e*., E_ssDNA/ssRNA-Prot_) as a function of *D*_*Conf*_; Fig 6) shows that the systems are sensitive to the ssDNA sequences. The overall shape of the energy landscape, as well as its high-density regions, change with altered sequences.

Fig 6 shows the binding pattern for three sequences: the wild-type (left), a shuffled sequence showing inferior binding compared with the wild-type (middle), and another shuffled sequence with better binding (right) (binding energies of additional ssDNA sequences are shown in S6 Fig). We note that, for the two telomeric ssDNA binding systems, Pot1pc (4HIO) and Cdc13 (1S40), the wild-type sequence tends to show better binding behavior compared with most of the shuffled sequences; the minimum energy structure of the wild-type sequence corresponds to the near-native structure. However, in both the cases, there are ssDNA sequences that show better binding behavior in terms of their similarity with the native structure as well as binding energy. The calculated binding energy for shuffled sequences demonstrate that the specific positions of ssDNA bases with respect to the aromatic residues (e.g., interactions between Trp and T or between Phe/Tyr with C, see Fig 1A) dictates the binding specificity for heterogeneous sequences for Pot1pc. The effect of sequence shuffling is weaker for Cdc13 that lacks any Trp at the interface. These observations suggest that base-mediated stacking interactions are critical for DNA specificity and that modeling enables a reliable prediction of the binding sequence to some extent. However, other factors, such as the rigidity/plasticity of the protein interface and the flexibility of the ssDNA and/or the protein may also play a role in sequence-specific binding. As such, sequence-specific protein–ssDNA interactions are achieved through a subtle balance of intermolecular interactions and dynamics.

### Binding energy: Electrostatic and aromatic contributions

To examine the role played by the electrostatic and aromatic interactions in the stability of the binding interface, we analyzed the energetics of the interfaces of the 12 studied ssDNA–ssDBP and ssRNA–ssRBP complexes. These structures bind their ssDNA/ssRNA ligands in three different ways that can be found in the following representative systems. i) The Cold shock protein from *Bacillus subtilis* (*i*.*e*., Bs-Csp; an OB fold), in which the ssDNA binding is largely mediated by base–aromatic interactions and the ssDNA backbone remains solvent exposed. ii) The human Poly(rC)-binding protein 1 (a KH fold), in which the ssDNA binds solely by electrostatic interactions using its phosphate backbone with no known instances of intermolecular aromatic interactions. iii) Telomere proteins (an OB-fold), which are known for sequence-specific DNA binding and the human hn-RNP A1 (RRM fold), where both electrostatic and aromatic energies are utilized to achieve specific binding. The contribution of the total electrostatic and aromatic energies is estimated by their ratio λ [= (total electrostatic energy)/(total aromatic energy)] calculated for the near-native structures (see Table 1).

A very low λ (<<1)for the B protein of Bs-Csp (Bs-CspB) indicates the importance of aromatic interactions for this protein, with this also clear from the predicted structures in Fig 4, where all ssDNA bases face toward the protein. By contrast, a very high value of λ (>>1)is obtained for the KH domain, indicating the importance of its electrostatic interactions; again, the representative structures in Fig 4 demonstrate that most of the DNA bases face away from the protein. The experimental structures of three other OB-folds, as well as the RRM domain, reveal that the ssDNA is oriented such that most of the bases face toward the protein surface to participate in stacking interactions, whereas electrostatic interactions with the phosphate backbone make a smaller contribution.

In the coarse-grained model, the contribution of the aromatic energy to the stability of the interface between ssDNA and the telomeric proteins was 2–4 times higher than the contribution of the electrostatic energy (*i*.*e*., 0.2>λ>0.4, see Table 1 and S7 Fig). Depending on the function of the ssDBP, different ssDBPs utilize different proportions of interactions in order to bind sequence-specifically or indiscriminately to their ssDNA ligands. Some of them interact with ssDNA largely by contacting the bases, whereas others minimize sequence specificity by controlling base stacking and base-specific H-bond formations, both of which might confer specificity.

Most of the ssRNA–ssRBP interfaces also reflect the importance of the aromatic interactions for their stability, as illustrated by the λ values being lower than 1. In these cases, the electrostatic interactions between the phosphate backbone and positively charged residues make a modest contribution to binding affinities. Similarly to the interaction of the cold shock protein with ssDNA, its interaction with ssRNA is also characterized by a very low λ value, showing that it is mediated by stacking interactions only; the corresponding predicted structure in Fig 4 also shows all RNA bases facing towards the protein. For the Fab structure, the RNA is recognized mostly by base–aromatic interactions mediated by a number of Tyr residues from the CDR region, and the estimated value of λ for this structure is also low. By contrast, λ>1 in the case of RAMP protein indicates the importance of the electrostatic contribution for the corresponding RNA binding. Indeed, here the ssRNA binds to the positively charged groove on the protein surface and electrostatic interaction plays a major role in binding.

### Estimated ssDNA/ssRNA–protein binding affinities correlate well with experimentally determined dissociation constant

**Fig 7:**
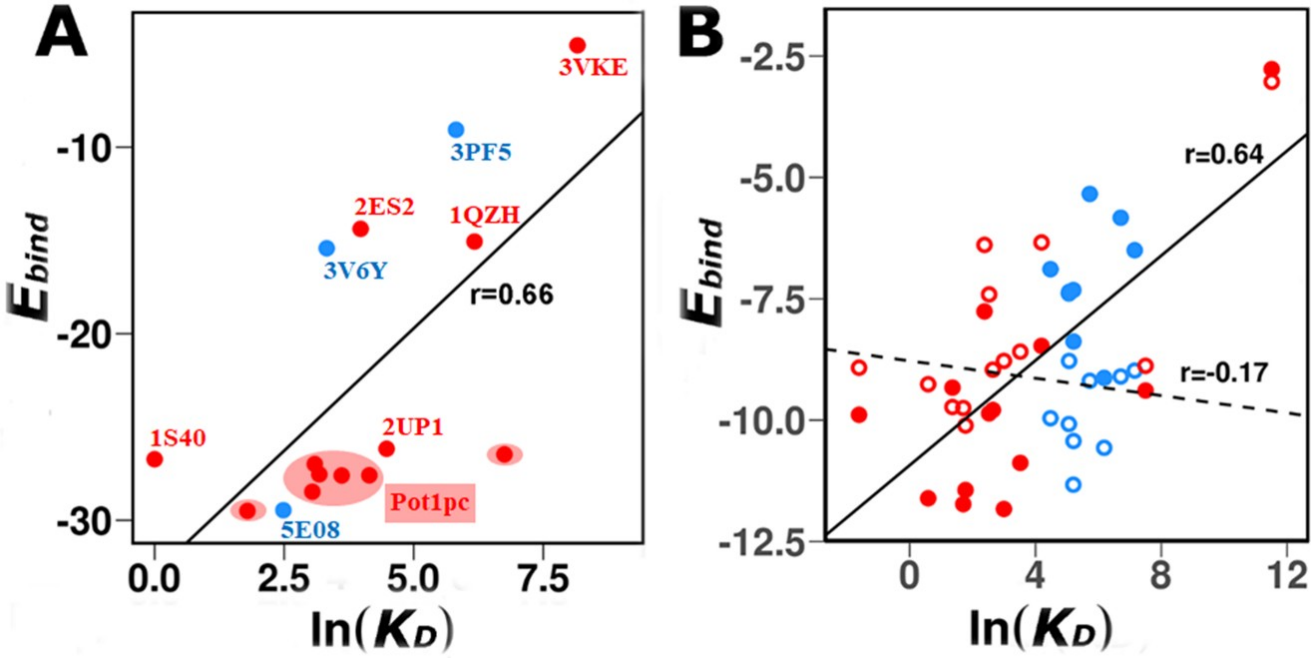
Correlation between estimated average binding energies (E_bind_, kcal mol^-1^) and the experimental binding free energies (ln(K_D_), where K_D_ is in nM). (a) Data for 12 different ssDBP–ssDNA (red) and three different ssRBP–ssRNA (blue) systems whose structures and dissociation constants are known. (b) Data for the cold-shock protein (13 ssDNA ligands in solid red, and nine ssRNA ligands in solid blue). To test the effect of ligand flexibility on binding, the dihedral potentials of ssDNA and ssRNA were interchanged such that the ssRNAs become more flexible than the ssDNAs. Corresponding trend shows that their binding energies also interchanged such that ssRNAs could bind the protein tighter than ssDNAs, so depicting the key role of flexibility in protein–ssDNA/ssRNA interactions. The solid and dashed lines are the linear correlation between the calculated binding energy and the experimental K_D_ for the ssDNA/ssRNA with the original or interchanged flexibility, respectively (with correlation coefficients of 0.64 and -0.17, which were obtained after excluding the data point of low affinity). For each ligand, the average binding energy was calculated by considering only those conformations that are similar to the experimental structure ($D_{Conf}^{1}$ and $D_{Conf}^{2}$ ≤5 Å). The trend line and corresponding Pearson correlation coefficients are reported.

In addition to the structural evaluation of the simulated binding of DBPs and RBPs with ssDNA or ssRNA, respectively, we were motivated to quantify the energetics of the predicted complexes. The binding energies, E_bind_ (*i*.*e*., E_ssDNA/ssRNA-Prot_) of the simulated complexes were compared with the experimentally measured equilibrium dissociation constants (*K*_D_). Fig 7A shows a comparison between E_bind_ and ln(*K*_D_) for different oligonucleotide sequences that bind six ssDBP and three ssRBPs. For Pot1pc, we calculated *E*_*bind*_ for seven different ssDNA sequences for which structures are available[78]. Overall, *K*_*D*_ values are in good agreement with *E*_*bind*_ (r = 0.66) indicating that the model captures the energetics of interaction between various proteins and ssDNA as well as ssRNA. In each case, E_bind_ was calculated by considering only near-native conformations ($D_{Conf}^{1}$ and $D_{Conf}^{2}$ ≤5 Å). Table 3 shows the *K*_*D*_ and E_bind_ values for each system.

**Table 3:** Experimental dissociation constants and calculated binding energies of different protein complexes with ssDNA or ssRNA.

ssDNA sequence	K_D_ (nM)	Calculated binding energy (kcal/mol)	ssRNA sequence	K_D_ (nM)	Calculated binding energy (kcal/mol)
**CspB**			**CspB**		
TTTTTT	1.8	-11.6 ± 2.2	UUUUUU	336	-9.1
CTTTTTT	5.9	-11.4 ± 2.5	GUCUUUU	88	-6.9 ± 2.0
CTTTTTC	33.7	-10.9 ± 2.5	GUCUUUA	159	-7.4 ± 2.4
CTCTTTC	3.9	-9.3 ± 2.5	GUCUUUG	158	-7.4 ± 1.6
CTCTCTC	10.8	-7.8 ± 1.9	AUCUUUG	485	-9.1 ± 2.5
CTCTTCC	66.2	-8.5 ± 2.0	CUCUUUG	822	-5.8 ± 1.7
CTCCTTC	12.5	-9.9 ± 1.5	UUUUUUU	183	-8.4 ± 2.2
CCCTTTC	1808	-9.4 ± 1.0	AGUUUUC	182	-7.3 ± 1.3
CCCCCCC	100000	-2.8 ± 1.5	UUCGUCU	1280	-6.5 ± 1.5
TTTTTTC	5.5	-11.7 ± 2.6	GUCUUGA	307	-5.3 ± 1.4
TTCTTTT	0.2	-9.9 ± 2.6	GUCUUUU	88	-6.9 ± 2.0
GTCTTTA	14.1	-9.8 ± 2.5			
TTTTTTT	1.8	-11.6 ± 2.2			
TTATTAG	20	-11.8 ± 2.3			
**hnRNP A1**			**Fab**		
TAGGGTTAGGG	88	-26.2	GUAUGCAUAGGC	12	-29.5
**Pot1pc**			**PUF**		
GCTTACGGT	855	-26.5 ± 2.1	CUGUGCCAUA	27.7	-15.4
GGATACGGT	37	-27.6 ± 2.4			
GGTAACGGT	21	-28.5 ± 2.9			
GGTTTCGGT	63	-27.6 ± 2.6			
GGTTAGGGT	6	-29.5 ± 2.4			
GGTTACGCT	22	-27.0 ± 2.1			
GGTTACGGT	24	-27.5 ± 2.6			
**Cdc13**					
GTGTGGGTGTG	1	-26.7			
**Pot1p**					
GGTTAC	480	-15.1 ± 2.6			
GGCTAC	High	-14.3 ± 2.2			
GGTCAC	High	-13.2 ± 2.6			
**Poly(rC)-binding protein 1**					
ACCCCA	3500	-4.5			

To compare E_bind_ with *K*_*D*_ for a particular protein that binds different ssDNA or ssRNA sequences, we analyzed the binding of Bs-CspB with various sequences of ssDNA and ssRNA. Two of its crystal structures were solved, one in complex with hexa-Thymine (dT_6_) (2ES2.pdb) that binds with nM affinity, the other one with hexa-Uracil (dU_6_) (3PF5.pdb), whose binding is weaker than that of dT_6_ but nevertheless in the nM range_._ The Bs-CspB binding site can interact with six to seven nucleotides[37]. The nucleic acid strands bind at the same binding site in the two structures, but their conformation differs at the 3’ end. Further investigations were also made on the binding affinities of Bs-CspB to different hepta-nucleotide ssDNA and ssRNA sequences that bind with a 1:1 stoichiometry [38]. In the crystal structure, several aromatic and hydrophobic solvent-exposed residues surrounded by basic side-chains form an amphiphilic surface that associates with the ligand. On the opposite surface, the protein comprises several acidic residues that impart a negative potential to the surface, making it unfit to bind either ssDNA or ssRNA. Moreover, the 0.88 Å Cα RMSD of this structure from free Bs-CspB (1CSP.pdb) shows a marginal conformational change of the protein due to ligand binding. Combining these observations, it is clear that Bs-CspB contains only a single binding site to which all the ssDNAs with variable sequences bind. Hence, in our analysis, we considered the bound conformation to be the same as that of Bs-CspB-dT_6_ for all ssDNA sequences. Starting from dT_7_, T was progressively replaced by C to investigate their preferences at each position, as was tested experimentally. The binding constant *K*_D_ of the resulting sequences varied in the μM to nM range, showing a preference for poly-Thymine over poly-Cytosine. Likewise, for all nine ssRNA sequences, the bound conformations were considered to be the same as in the Bs-CspB.dU_6_ complex. The binding constant of ssRNAs also varied in the μM to nM range, however the values were lower than for ssDNAs.

The E_bind_ versus *K*_*D*_ plot for 13 ssDNA (solid red circles) and nine ssRNA (solid circles, blue) that bind to Bs-CspB is shown in Fig 7B. Overall, they are in good agreement with a linear fit (R = 0.76, considering solid circles only). Our model captures the overall higher affinity of Bs-CspB for ssDNA compared with ssRNA. Similarly to the experimental data, the binding energies (E_bind_) of the polythymine and polycytosine sequences in the coarse-grained model indicate that the former is more stable. However, the E_bind_ was less sensitive in predicting the effect on *K*_D_ of a single mutation at different positions. This is expected, as achieving such accuracy is beyond the scope of any coarse-grained model. Nonetheless, results from our simulations agreed well with the experimental binding affinities when nucleotide content was taken into account, and thus such simulations can be used in binding specificity predictions.

To understand the origin of the higher affinities of Bs-CspB for ssDNA than for ssRNA, we used the simulated binding events to estimate the association and dissociation rates for the interactions of the GTCTTTA ssDNA sequence and GUCUUUA ssRNA sequence with the cold shock protein, for which experimental kinetic results are available[38]. Computationally, the rate constant for association (*k*_*on*_) was estimated by the elapsed time for binding (defined by *D*_*conf*_ <5Å) when starting from an unbound state, and similarly the rate constant for dissociation (*k*_*off*_) was estimated by the elapsed time for dissociation (defined by *D*_*conf*_>5Å) when starting from the bound complex. The association constant ratio *k*_*on*_(ssDNA)/*k*_*on*_(ssRNA) from the coarse-grained simulations is ~1, in very good agreement with the experimental data. The dissociation constant ratio *k*_*off*_(ssDNA)/*k*_*off*_(ssRNA) from the simulations is ~0.2. The value of this ratio based on the experimental results is 0.1[38], yet both the simulations and the experimental data agree that the ratio is lower than unity. The higher dissociation rate for ssRNA compared with ssDNA is the main reason for the higher K_D_ of Bs-CspB–ssRNA compared with Bs-CspB–ssDNA.

### Flexibility of ssDNA and ssRNA and their role in binding

The energy contribution from electrostatic and aromatic interactions plays a significant role in ssDNA/ssRNA binding with proteins. Nevertheless, it is not only the charged or aromatic side-chains that interact with the nucleic acid backbone or bases, respectively, to govern the protein–ssDNA/ssRNA assembly. For example, some charged residues that do not interact directly with DNA or RNA can still have a strong electrostatic effect on binding [80,81]. Unbound ssDNAs/ssRNAs are highly flexible in solution, without any definite shape. Prior to binding, they fluctuate in an ensemble whose length and shape match the size of the binding pocket. Their conformational flexibility usually leads to an induced fit of the ssDNA/ssRNA to the protein surface. Complexes between ssDNA/ssRNA and protein are therefore difficult to predict unless their backbone flexibility is properly modeled. In our model, we focused on incorporating the conformational flexibility of the ssDNA and ssRNA.

The flexibility of ssDNA or ssRNA is often judged by their persistence length, where a lower persistence length value corresponds to greater flexibility. The persistence length of both ssDNA and ssRNA decreases with increasing salt concentration[12]. However, when their persistence lengths are compared, ssDNA was found to have lower averages compared with ssRNA, which indicates that, in solution, ssDNAs are more flexible than ssRNAs. In our coarse-grained model, we mimicked the effect of salt concentration by means of dihedral potentials (see Methods), where the persistence length of ssDNA/ssRNA in solution increases with increasing dihedral potentials and decreases with increasing salt concentration[12]. To be consistent with the experimental finding, we set the dihedral potentials in the model such that ssDNA possess a lower persistence length (greater flexibility) than ssRNA.

Further to compare the role of flexibility for ssDNA and ssRNA in their differential binding strengths, we used the Bs-CspB model system for which binding data for a number of ssDNA as well as ssRNA molecules are available. The dihedral parameters of ssDNAs and ssRNAs were interchanged so that ssRNAs became more flexible than ssDNAs. All other parameters including base–aromatic stacking strengths were unaltered. The resulting ssRNAs (Fig 7B, empty red circles) were found to bind Bs-CspB more tightly than ssDNAs do (Fig 7B, empty blue circles). The correlation between the binding energy of the modified ssDNA and ssRNA, in which their degree of flexibility was switched, and the experimental *K*_*D*_ values is much weaker (Fig 7B). This observation indicates the major role that flexibility plays in their binding. It can further explain why ssDNAs–protein interactions can be stronger than ssRNA–protein interactions.

### Binding specificity of ssDNA and ssRNA with their protein partners

Often, biomolecular affinity and specificity are linked and they can also be related to the degree of flexibility of the ligand[82–85]. Although, conventionally, high affinity is linked with high specificity, there are examples of flexibility resulting in reduced affinity while high specificity is retained. The interactions of ssDNA and ssRNA with their protein receptors are shown to differ with respect to their affinity (Fig 7B). This, together with their different conformational flexibilities, may suggest that they may have different degrees of specificity [82–85]. Specificity is often defined as the binding affinity to one ligand relative to other ligands. Alternatively, one may define the intrinsic specificity, which is the binding affinity of a ligand to a receptor relative to the binding affinity of the ligand to other sites on the same receptor[86].

To quantify the decoupling between the affinity and specificity of ssDNA/ssRNA binding to proteins, and the link to their different intrinsic flexibilities, we analyzed the energy landscape for binding using the theory of energy landscape [87–90]. According to this theory, the native conformation of the binding complex is the conformation with the lowest binding energy and the energies of the non-native conformations follow a statistical Gaussian distribution. A dimensionless quantity termed the intrinsic specificity ratio (ISR) is defined to describe the magnitude of intrinsic specificity [86,91,92]: $ISR=\delta E/(\Delta E\sqrt{2S})$, where δE is the energy gap between the native binding state and the average non-native binding states, ΔE is the energy variance of the non-native states, and *S* is the configurational entropy. A large ISR value indicates that the protein strongly discriminates the native binding site from the non-native binding sites, which indicates a high binding specificity.

**Fig 8:**
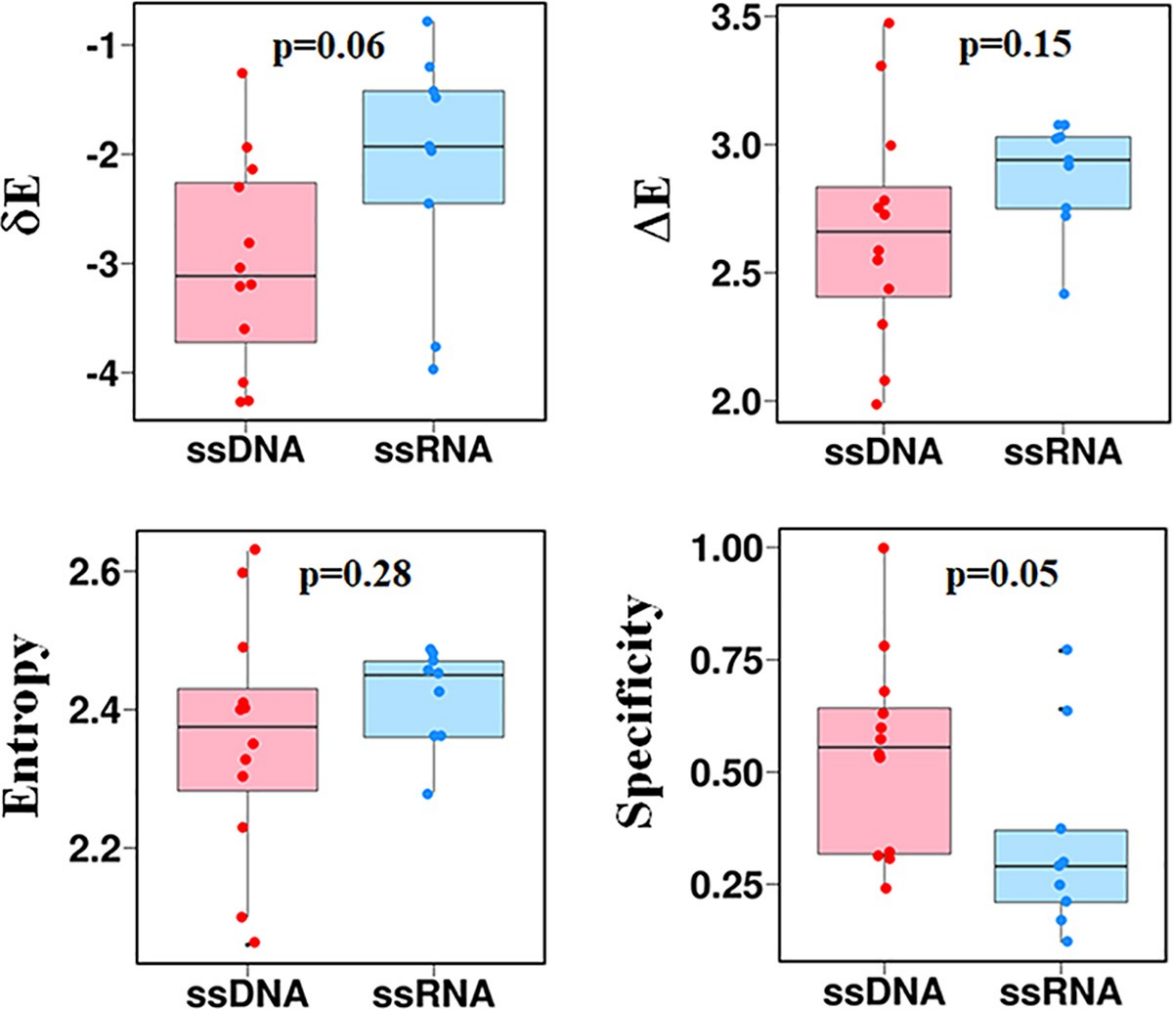
Binding specificity of ssDNA *vs*. ssRNA. To judge the specificity with which different sequences of ssDNA and ssRNA bind to the cold shock protein (2ES2.pdb and 3PF5.pdb, respectively), the intrinsic specificity ratio, ISR = $\frac{\delta E}{\Delta E\surd 2S}$, was calculated. The *δE* term represents the energy gap between the native binding state (average binding energy of all near-native structures from the simulation obtained by applying the condition $D_{Conf}^{1},D_{Conf}^{2}$≤3Å) and the average non-native binding states (average binding energy of all non-native binding conformations from the simulation), *ΔE* is the energy variance of the non-native states and *S* is the entropy of binding energy. The non-native interactions are defined as conformations that have low binding energy (i.e., a negative binding energy) and that satisfy $D_{Conf}^{1},D_{Conf}^{2}$>3Å). The entropy is estimated as *Σplog(p)*, where *p* is the probability of having non-native conformation with a certain binding energy obtained by binning. Panels show the values of *δE*, *ΔE*, *S*, and specificity for complexes of the cold shock proteins with ssDNA (red) and ssRNA (blue). A higher energy gap, lower energy variance, and slightly lower entropy were found for ssDNAs compared to ssRNAs. Thus, compared with their ssRNA counterparts, ssDNAs are found to have a higher binding specificity.

The energy landscapes for the association of twelve ssDNA and nine ssRNA sequences with their corresponding protein receptors were analyzed by estimating the values of *δE*, *ΔE*, and *S*. Fig 8 shows that the complexes formed with ssDNA have smaller *δE* and *ΔE* values than the complexes with ssRNA. Namely, the native complexes of ssDNA–ssDBP are more distinguished energetically than the non-native conformation in comparison to the ssRNA–ssRBP complexes. Furthermore, on average, the non-native ssDNA–ssDBP complexes are less diverse than the ssRNA–ssRBP complexes. These two properties and their similar entropy, *S*, result in higher specificity for the ssDNA complexes than for the ssRNA complexes. In summary, the differences between the ssDNA–ssDBP and ssRNA–ssRBP complexes are due to the greater flexibility of ssDNA compared with ssRNA, which leads to higher affinity (Fig 8B) and higher specificity.

## Discussion

Predicting the complexes formed between proteins and either ssDNA or ssRNA is difficult because of their complex underlying energy landscapes, which originate mostly from the considerable flexibility of the ssDNA or ssRNA and their consequent lack of a defined structure. Effective factors that can be tuned to affect the interaction between single-stranded nucleic acids and receptor proteins include the extent of ligand flexibility and also the salt concentration, which may modulate the strength of the electrostatic interactions. The lack of an ordered structure in ssDNAs or ssRNAs allows them to interact with proteins not only through electrostatic interactions between their backbone phosphate groups and positively charged residues but also by stacking interactions between free nucleotide bases and aromatic side chains. These factors increase the degree of complexity and heterogeneity of these interfaces and thus computational modeling of specific ssDNA–ssDBP and ssRNA–ssRBP interactions becomes even more challenging compared with other specific macromolecular interactions. As a result, unlike protein–protein or protein–dsDNA interactions, the theoretical study of ssDNA/ssRNA–protein binding specificity from the structural and energetic points of view is not sufficiently advanced.

In this study, we applied a physically based coarse-grained approach to construct a generalized model to study the recognition of ssDNA/ssRNA by ssDBPs and ssRBPs, respectively. A number of experimental studies showed that there are no obvious structural indicators for sequence-specific proteins [37,40,78]. Instead of strictly binding or not binding to particular sequences, a protein can bind different sequences with a range of affinities. From the perspective of structural properties, specific binding can be attributed to specific base–aromatic interactions and to the ssDNA/ssRNA dynamics. We incorporated binding specificity into the model by adding different base–aromatic stacking strengths as well as by adjusting the flexibility of the single-stranded nucleic acid. Accordingly, the model has only two free parameters.

The developed transferable coarse-grained model was successfully applied to 12 complexes between ssDNAs or ssRNAs and binding proteins. The results demonstrated that single stranded nucleotide–protein recognition follows the binding energy model in which the predicted near-native structures correspond to minimum binding energies. The predicted complexes differ in the relative energetic contributions made to them by aromatic and electrostatic interactions. Few interfaces are governed solely by either electrostatic or aromatic interactions, rather, the majority of the interfaces are stabilized by both electrostatic and aromatic interactions, with the latter being more dominant. The model is sensitive to sequence-specific binding and the estimated interfacial binding energies of near-native conformations show good correlation with experimental dissociation constants.

Our results suggest that the origin for the weaker stability of the complexes formed between proteins and ssRNA compared with ssDNA is the lower flexibility of ssRNA. The lower affinities of ssRNA–ssRBP compared with ssDNA–ssDBP are coupled with larger dissociation rate constants (*k*_*off*_) while their association rate constants (*k*_*on*_) are of similar values. The complexes of ssRNA are also found to be less specific than those of ssDNA, which might be linked to their greater stiffness.

While the power of the developed coarse-grained model lies in its simplicity, which allows extensive sampling of several systems and thus enables the study of long timescale dynamic motions, it can be further advanced to address other molecular biophysical aspects of protein–ssDNA/ssRNA dynamics. For example, incorporating specific and explicit ion interactions with ssDNA and ssRNA and their interactions with the solvent may improve the accuracy of the predicted structures. Sequence specificity may also depend on base-specific hydrogen bonding networks that are formed between the single stranded nucleic acids and the proteins, implementation of which would enhance the efficiency of the model for specific recognition. Furthermore, the model deals with unstructured ssDNA and ssRNA and it may demand additional energetic terms to represent formations of more compact structures of ssDNA mediated by base-pairing and, in particular, the formation of secondary structures in ssRNAs. Nonetheless, the present model produces useful results for specific ssDNA–ssDBPs interactions, and thus this type of coarse-grained model can be further used to study other properties of these interactions (e.g., the sliding mechanism of ssDNA on ssDBPs; [93–95]), to complement experimental studies, and especially to elucidate how the molecular properties of the interfaces are linked to their function and dynamics.

## Supporting information

S1 Fig Conformational ensemble of predicted structures of proteins with single-stranded nucleic acids. The population distribution of predicted conformations is shown for ssDBP–ssDNA (top, red square) and ssRBP–ssRNA (bottom, blue square) complexes. The plots are similar to those presented in Fifure 2 but for six different ssDBP-ssDNA and ssRBP-ssRNA. Representative conformations from three regions marked 1–3 in the current figure are shown in S2 Fig. Additional molecular and structural details for each of the complexes can be found in Table 1.
(TIF)

S2 Fig Comparing the power of model of heterogeneous and homogenous ssDNA in predicting their complexes with telomeric proteins. The heterogeneous model refers to the model presented in the current manuscript and the homogenous (polyT) model refers to the model presented in ref. # 47. The number in the right-bottom corner of each panel corresponds to the percentage of native-like conformations (*D*_*1*_, *D*_*2*_ ≤ 5Å).
(TIF)

S3 FigThree representative conformations from simulations that correspond to the densely populated regions.The regions are labelled 1, 2, and 3 in S1 Fig are shown in green, red, and blue, respectively, for each of the ssDBP–ssDNA (top, red square) and ssRBP–ssRNA (bottom, blue square) complexes. All-atom cartoon representations of the protein (in gray) and of the bound conformation of the ssDNA or ssRNA (in orange) are shown for comparison. The lowest energy green ssDNA/ssRNA conformations (region 1) are most similar to the orange experimental conformations (lower values of $D_{Conf}^{1}$ and $D_{Conf}^{2}$ and of $D_{Site}^{1}$ and $D_{Site}^{2}$), which demonstrates the predictive power of the model.(TIF)

S4 Fig Accuracy of the predicted conformation of ssDNA/ssRNA backbone and bases. Group A and B corresponds to the backbone and base beads, respectively. This analysis was performed for the native-like conformations.
(TIF)

S5 Fig Energy landscapes for simulated ssDNA–ssDBP and ssRNA–ssRBP complexes (shown in S1 Fig). The binding energy (Kcal mol^-1^) is plotted versus *D*_*Site*_ and *D*_*Conf*_ for each of the ssDBP–ssDNA (top, red square) and ssRBP–ssRNA (bottom, blue square) complexes. The points encircled in green, red, and blue correspond to the respective ssDNA/ssRNA conformations shown in S2 Fig. The population density of the ssDNA/ssRNA ensemble is shown by orange contour lines. A funnel-shaped binding energy landscape is present in all cases, with ssDNA/ssRNA conformations closest to the experimental structures possessing the minimal energy.
(TIF)

S6 Fig Plots of calculated binding energy vs *D_Conf_* for shuffled sequences. The complexes between Pot1pc (4HIO) and Cdc13 (1S40) telomeric proteins and seven different sequences of ssDNA were studied. The energy plots demonstrate that the specific positions of ssDNA bases with respect to the aromatic residues (e.g., C base with Trp; TT base with Phe and Tyr) dictate the binding specificity for heterogeneous sequences. The effect of sequence shuffling is larger for 4HIO with ssDNA comprise all four nucleotides than the more homogeneous ssDNA sequences for 1S40 in which the interface also does not have any Trp.
(TIF)

S7 Fig The contribution of electrostatic and aromatic energies to the energy landscape of binding. The total binding energy (right column) is decomposed into aromatic energy (left column) and electrostatic energy (middle common) along *D*_*site*_. For most systems, the aromatic interactions govern the shape of the energy landscape for binding. The exceptional case is 3VKE that is stabilized by electrostatic interactions.
(TIF)
