# Supplementary figures and images for: Structure, stability and specificity of the binding of ssDNA and ssRNA with proteins
Source: PLoS Comput Biol. 2019 Apr 1;15(4):e1006768. doi: 10.1371/journal.pcbi.1006768 (PMC6467422; doi:10.1371/journal.pcbi.1006768)

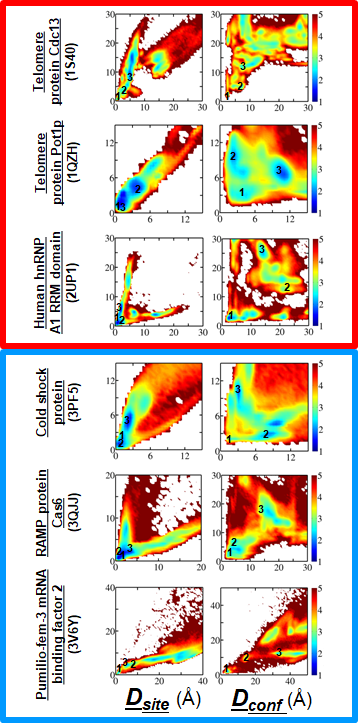

Supplement: S1 Fig — The population distribution of predicted conformations is shown for ssDBP–ssDNA (top, red square) and ssRBP–ssRNA (bottom, blue square) complexes. The plots are similar to those presented in Fifure 2 but for six different ssDBP-ssDNA and ssRBP-ssRNA. Representative conformations from three regions marked 1–3 in the current figure are shown in S2 Fig. Additional molecular and structural details for each of the complexes can be found in Table 1. (TIF) [file pcbi.1006768.s001.tif]

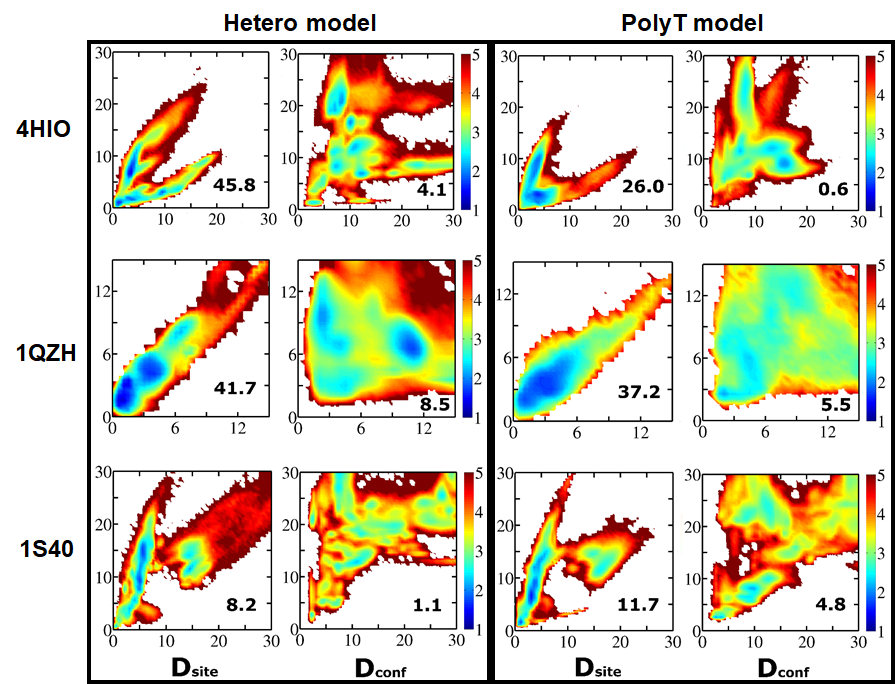

Supplement: S2 Fig — The heterogeneous model refers to the model presented in the current manuscript and the homogenous (polyT) model refers to the model presented in ref. # 47. The number in the right-bottom corner of each panel corresponds to the percentage of native-like conformations (D1, D2 ≤ 5Å). (TIF) [file pcbi.1006768.s002.tif]

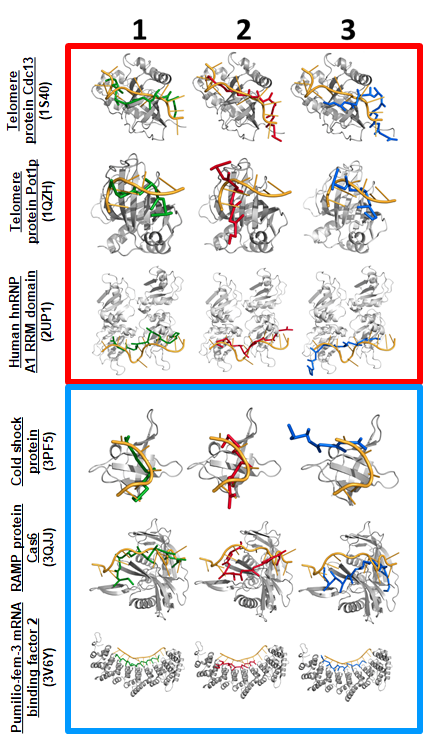

Supplement: S3 Fig — The regions are labelled 1, 2, and 3 in S1 Fig are shown in green, red, and blue, respectively, for each of the ssDBP–ssDNA (top, red square) and ssRBP–ssRNA (bottom, blue square) complexes. All-atom cartoon representations of the protein (in gray) and of the bound conformation of the ssDNA or ssRNA (in orange) are shown for comparison. The lowest energy green ssDNA/ssRNA conformations (region 1) are most similar to the orange experimental conformations (lower values of DConf1 and DConf2 and of DSite1 and DSite2), which demonstrates the predictive power of the model. (TIF) [file pcbi.1006768.s003.tif]

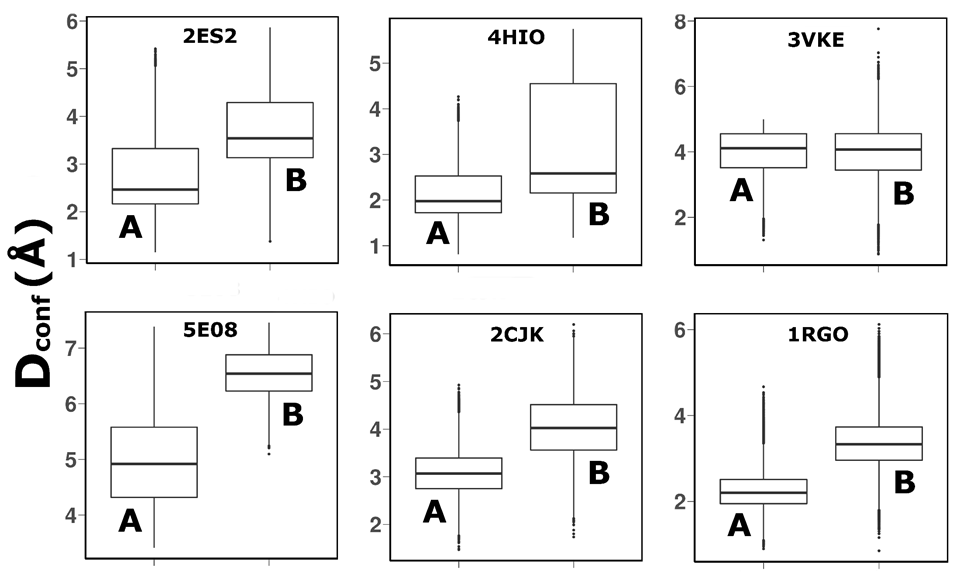

Supplement: S4 Fig — Group A and B corresponds to the backbone and base beads, respectively. This analysis was performed for the native-like conformations. (TIF) [file pcbi.1006768.s004.tif]

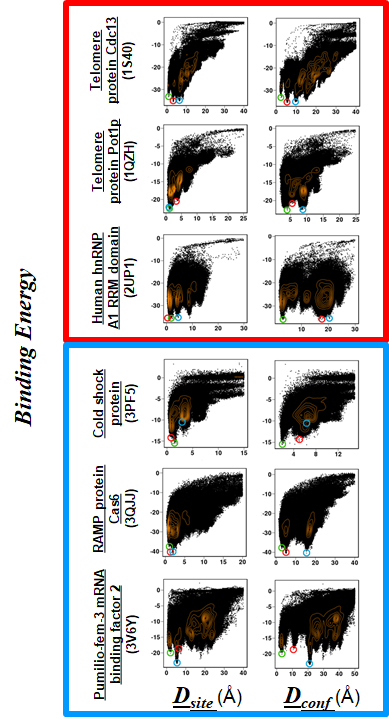

Supplement: S5 Fig — The binding energy (Kcal mol-1) is plotted versus DSite and DConf for each of the ssDBP–ssDNA (top, red square) and ssRBP–ssRNA (bottom, blue square) complexes. The points encircled in green, red, and blue correspond to the respective ssDNA/ssRNA conformations shown in S2 Fig. The population density of the ssDNA/ssRNA ensemble is shown by orange contour lines. A funnel-shaped binding energy landscape is present in all cases, with ssDNA/ssRNA conformations closest to the experimental structures possessing the minimal energy. (TIF) [file pcbi.1006768.s005.tif]

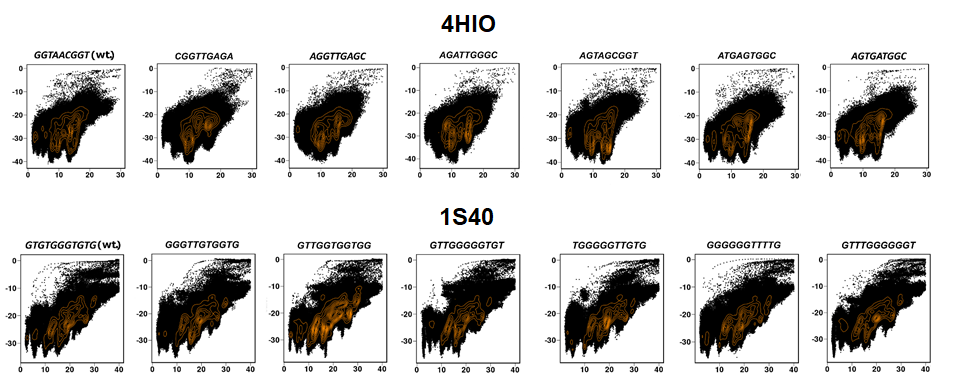

Supplement: S6 Fig — The complexes between Pot1pc (4HIO) and Cdc13 (1S40) telomeric proteins and seven different sequences of ssDNA were studied. The energy plots demonstrate that the specific positions of ssDNA bases with respect to the aromatic residues (e.g., C base with Trp; TT base with Phe and Tyr) dictate the binding specificity for heterogeneous sequences. The effect of sequence shuffling is larger for 4HIO with ssDNA comprise all four nucleotides than the more homogeneous ssDNA sequences for 1S40 in which the interface also does not have any Trp. (TIF) [file pcbi.1006768.s006.tif]

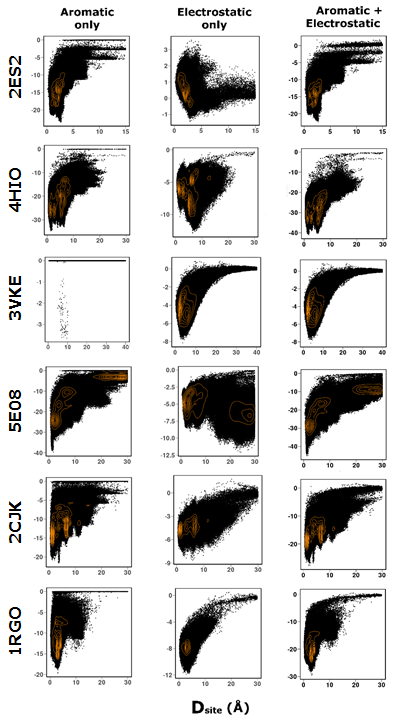

Supplement: S7 Fig — The total binding energy (right column) is decomposed into aromatic energy (left column) and electrostatic energy (middle common) along Dsite. For most systems, the aromatic interactions govern the shape of the energy landscape for binding. The exceptional case is 3VKE that is stabilized by electrostatic interactions. (TIF) [file pcbi.1006768.s007.tif]
